# Dynamics of acquisition and loss of carriage of *Staphylococcus aureus* strains in the community: The effect of clonal complex^☆^^☆☆^

**DOI:** 10.1016/j.jinf.2013.12.013

**Published:** 2014-05

**Authors:** Ruth R. Miller, A. Sarah Walker, Heather Godwin, Rowena Fung, Antonina Votintseva, Rory Bowden, David Mant, Timothy E.A. Peto, Derrick W. Crook, Kyle Knox

**Affiliations:** a Nuffield Department of Clinical Medicine, University of Oxford, John Radcliffe Hospital, Oxford OX3 9DU, United Kingdom; b UBC School of Population and Public Health, British Columbia Centre for Disease Control, 655 West 12th Avenue, Vancouver, BC V5Z 4R4, Canada; c National Institute for Health Research Oxford Biomedical Research Centre, John Radcliffe Hospital, Oxford OX3 9DU, United Kingdom; d Oxford University Hospitals National Health Service Trust, Oxford OX3 9DU, United Kingdom; e Department of Statistics, University of Oxford, Oxford OX1 3TG, United Kingdom; f Wellcome Trust Centre for Human Genetics, Oxford OX3 7BN, United Kingdom; g Department of Primary Care Health Sciences, University of Oxford, Oxford OX1 2ET, United Kingdom

**Keywords:** *Staphylococcus aureus*, Molecular epidemiology, Colonisation, *spa*-typing, Carriage duration

## Abstract

**Background:**

*Staphylococcus aureus* nasal carriage increases infection risk. However, few studies have investigated *S. aureus* acquisition/loss over >1 year, and fewer still used molecular typing.

**Methods:**

1123 adults attending five Oxfordshire general practices had nasal swabs taken. 571 were re-swabbed after one month then every two months for median two years. All *S. aureus* isolates were *spa*-typed. Risk factors were collected from interviews and medical records.

**Results:**

32% carried *S. aureus* at recruitment (<1% MRSA). Rates of *spa*-type acquisition were similar in participants *S. aureus* positive (1.4%/month) and negative (1.8%/month, *P* = 0.13) at recruitment. Rates were faster in those carrying clonal complex (CC)15 (adjusted (*a*)*P* = 0.03) or CC8 (including USA300) (*aP* = 0.001) at recruitment versus other CCs. 157/274 (57%) participants *S. aureus* positive at recruitment returning ≥12 swabs carried *S. aureus* consistently, of whom 135 carried the same *spa*-type. CC22 (including EMRSA-15) was more prevalent in long-term than intermittent *spa*-type carriers (*aP* = 0.03). Antibiotics transiently reduced carriage, but no other modifiable risk factors were found.

**Conclusions:**

Both transient and longer-term carriage exist; however, the approximately constant rates of *S. aureus* gain and loss suggest that ‘never’ or truly ‘persistent’ carriage are rare. Long-term carriage varies by strain, offering new explanations for the success of certain *S. aureus* clones.

## Introduction

*Staphylococcus aureus* is an important cause of infections in both primary and secondary care. Carriage prevalences of ∼30% have been found consistently in studies performed over six decades,^1^ with the anterior nares the primary site of colonisation.^1–3^ Nasal carriers are at greater risk of infection than non-carriers^4–7^ and the carried and invasive strains are indistinguishable in ∼80% of cases.^5,8^ Non-carriers of *S. aureus* have a higher mortality following *S. aureus* bacteraemia suggesting recent *S. aureus* acquisition around the time of infection is associated with poorer subsequent outcome.^5^

The dynamic nature of *S. aureus* carriage creates complexity for cross-sectional and longitudinal studies, with people acquiring and losing all genotypes of *S. aureus* (the species level) and also acquiring and losing different genotypes within *S. aureus*.^9^ For example, one study found multiple genotypes were present in 7% of carriage samples.^10^

Rather than considering *S. aureus* loss and acquisition as separate events, studies have almost universally combined both these aspects and classified individuals as “persistent”, “intermittent” or “non” carriers. “Persistence” has most commonly been defined on the basis of (i) >80% positivity of 10–12 swabs taken weekly over ∼3 months (not considering strain-type)^11^ or (ii) two positive cultures one week apart, since this had good performance for predicting persistent carriage defined by definition (i) in one study.^12^ Human polymorphisms associated with “persistent” carriage using definition (ii) have been identified,^13^ but bacterial factors have not, to date, been associated with different carriage types. Very long-term carriage and strain switching undoubtedly occur; for example 12/17 “persistent” *S. aureus* carriers according to definition (i) carried *S. aureus* on a single swab taken eight years later, but only three carried highly similar *S. aureus* strains.^11^ However, few studies appear to have repeatedly sampled individuals over intermediate periods of >1 years,^14,15^ or systematically investigated carried genotypes over these timescales. The rates of acquisition and median carriage duration of newly acquired strains, and the rates of loss of individual strains present in an initial sample with unknown acquisition date, have also rarely been described outside the specific setting of methicillin-resistant strains in hospitalised patients.^16–18^ Longer-term follow-up might further support experimental studies which found no distinction between non- and intermittent carriers defined following definition (i) in terms of rates of loss of carriage of a nasal inoculum.^19^

Here we investigate *S. aureus* nasal carriage in individuals from primary care, swabbed bi-monthly for up to 36 months. We *spa*-typed all *S. aureus* isolates to identify acquisition and loss that would be unrecognised at the species level. Our primary objective was to describe the dynamics of *S. aureus* carriage (loss, gain) in the general population, and to investigate potential risk factors, in particular the contribution from particular *spa*-types.

## Methods

### Study population

Eligible participants were consecutive adults aged ≥16 years attending one of five Oxfordshire general practices (each a group of family doctors) in the Thames Valley Primary Care Research Partnership (all in the catchment area for the Oxford University Hospitals (OUH) NHS Trust). All participants provided written informed consent. 200 participants were recruited from each general practice sequentially over December 2008–December 2009, in age/sex strata approximately representing the UK population. Recruitment was completed in each practice before starting in the next. To increase numbers of younger participants, students registering at one practice were recruited during the University Freshers' week. For the first four general practices, we invited only those participants whose recruitment swab grew *S. aureus* to continue longitudinal follow-up. All participants from the last practice and all students were invited to continue longitudinal follow-up. Assuming 35% participants were *S. aureus* positive at recruitment and followed longitudinally, recruiting 1000 adults provided >80% power to detect differences of >15% in risk factors between intermittent and continuous carriers.^17,20–22^ The study was approved by Oxfordshire Research Ethics Committee B (08/H0605/102).

Nasal swabs were taken by all participants on recruitment under research nurse supervision. A dry cotton swab was placed in the tip of both nostrils and rotated three times. All *S. aureus* positive participants, all students and all participants from the last practice were posted a nasal swabbing kit one and two months after recruitment, and then every two months thereafter. The swabbing technique was demonstrated on recruitment and explained in a leaflet included with each kit. Swabs were returned by post in charcoal medium (typically <1 week), and stored at 4 °C on receipt before processing (processing took <1 week; up to two weeks in total). As the study objective was to investigate *S. aureus* dynamics, isolation protocols focussed on identifying all strains, even those present at low frequencies. To increase the sensitivity of culture, swabs were therefore incubated overnight at 37 °C in 5% NaCl enrichment broth (E&O Laboratories, Bonnybridge, UK). A 5 mm loop-full of broth was sub-cultured onto SASelect^®^ chromogenic agar (Bio-Rad, Limerick, Ireland) and incubated at 37 °C overnight. Pink colonies were tested further using DNAse, catalase and Staphaurex tests following standard procedures.^23^ Samples positive in all three tests were presumed to be *S. aureus*. A selection of pink colonies from the SASelect agar were resuspended in saline from which one aliquot was stored as glycerol stock at −20 °C and another added to 10 μl 0.85% Saline (E&O Laboratories) and 50 μl TE buffer (Sigma, Dorset, UK), heated at 99.9 °C for 10 min, then centrifuged to separate the supernatant. From this, 50 μl was removed and stored at −20 °C as a crude chromosomal DNA extract.

*spa*-typing was performed as described,^24^ with DNA amplification and sequencing using the Microlab Star Liquid Handling Workstation (Hamilton Robotics Ltd, Birmingham, UK). Chromatograms for the *spa* gene were assembled using Ridom StaphType.^24^ Samples with mixed chromatograms were re-cultured and six-12 colonies separately typed. *spa*-types were grouped into *spa*-Clonal Complexes (CCs) using BURP clustering, and CCs labelled as their MLST equivalent for ease of comparison with other studies.^25^

Epidemiological and healthcare information was collected from a structured questionnaire at recruitment, general practice and OUH records (see Supplementary Methods). After two years follow-up, general practice and OUH records were re-reviewed to ascertain antimicrobial use and inpatient admissions throughout follow-up.

### Outcome definitions

(1) Loss of carriage (primary outcome)

Confirmed loss of carriage was defined as two consecutive negative swabs (or two consecutive swabs without the previous *spa*-type for analysis of *spa*-types (*spa*-level)). The time of confirmed loss was taken as the first of the two negatives. Single isolated negatives were ignored (given potential limited efficacy of self-swabbing). Participants with only their last swab negative were censored at the preceding positive swab. Thus loss analyses included only participants returning ≥2 swabs after the first positive to enable any loss to be confirmed. Loss rates over time were estimated using flexible parametric hazard models.^26^

(2) Acquisition

*S. aureus* acquisition was defined as positive growth (or a new *spa*-type) after confirmed prior absence (two consecutive negatives, or absence of *spa*-type). Thus if the first swab after recruitment (post-recruitment swab) in individuals *S. aureus* negative at recruitment (recruitment-negatives) grew *S. aureus* (or grew a new *spa*-type in participants *S. aureus* positive at recruitment (recruitment-positives)), this was not counted as acquisition but was presumed to represent a false-negative result at recruitment. Acquisition analyses therefore also included only participants returning ≥2 swabs after the first positive. Since nasal evolution can produce small changes in repeat numbers, new *spa*-type acquisition was defined as having >2 differences from first positive swab^25^ (see Supplementary Table 1 for grouping). Results were similar allowing any *spa* difference to count as a new *spa*-type acquisition.

All individuals were to be followed for two years (total 14 swabs) under the original protocol. If an individual did not return three consecutive swabs, no further swabs were sent. Following a protocol amendment, at two years further consent was sought for longer follow-up in those persistently negative or persistently positive (allowing single intermitted negatives) for *S. aureus* to enable longer-term rates of gain and loss to be estimated in those remaining at risk. Participants were considered formally lost to follow-up if they returned their last swab <22 months from enrolment and >4 months before the data cut-off (23 January 2012).

### Statistical analysis

STATA 11.2 was used for all analyses, which included data to 23 January 2012 (minimum two years expected follow-up). Cox regression was used to identify independent predictors of loss and acquisition. Multinomial logistic regression was used to identify predictors of long-term carriage with the same *spa*-type versus intermittent carriage, and predictors of never observed versus intermittent carriage (see Supplementary Methods). The modal CC was defined as the most frequently observed CC per individual.

## Results

### *S. aureus* at recruitment

Of 1123 enrolled individuals, 360 (32%) were *S. aureus* positive at recruitment. Four hundred and eighty-three (43%) were male and the median age of all 1123 individuals was 55 (inter-quartile range (IQR)) [range] (37–67) [16–94] years. Nine individuals had MRSA (0.8%) at recruitment, all Epidemic-(E)MRSA-15 (*N* = 3) or EMRSA-16 (*N* = 6). Male sex, being in current employment, longer time since district nurse appointment, ever having been an inpatient and treatment for a skin condition within the last 30 days were associated with higher *S. aureus* prevalence in a multivariate model (*P* < 0.0001, 0.02, 0.04, 0.03, 0.03 respectively) (Supplementary Table 1).

### *S. aureus* carriage during follow-up

**Supplementary Figure 1:**
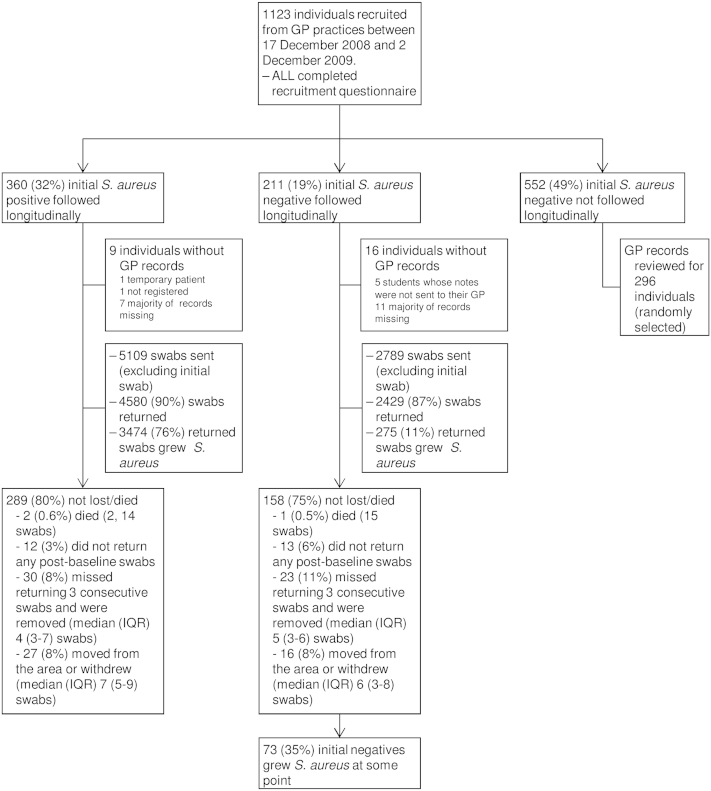
Flow diagram of participants followed up. *Note*: when the first *S. aureus* positive swab contained multiple *spa*-types, time to loss of *spa*-type from recruitment swab was the first time any of these *spa*-types was lost.

**Figure 1:**
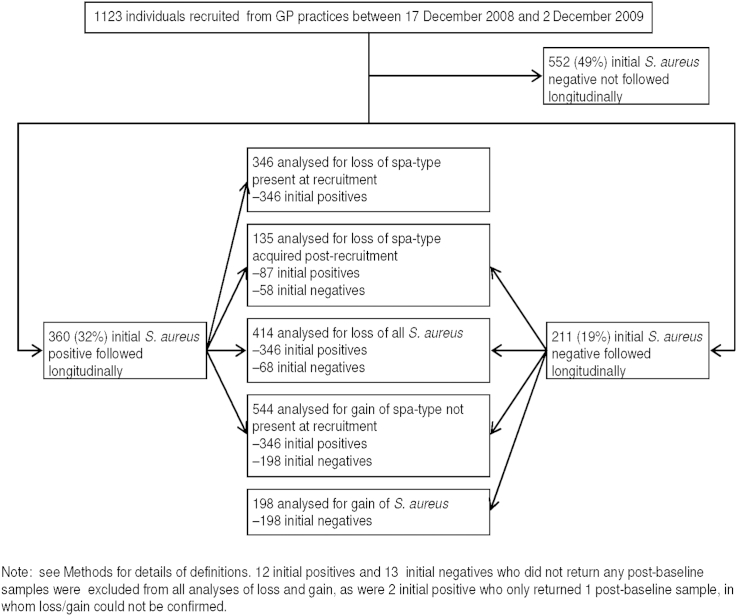
Study flow diagram.

To investigate *S. aureus* loss and (re-)acquisition, the 360 individuals positive at recruitment (recruitment-positive) plus a further 211 *S. aureus* negative at recruitment (82 from the last general practice, 129 students, see Methods) were followed for a median (IQR) 2.0 (1.8–2.2) years, returning a median (IQR) 14^11–15^ swabs (range 1–20). Three (0.5%) individuals died and 121 (21%) were lost to follow-up (25 (4%) did not return any swabs post-baseline, 53 (9%) missed returning three consecutive swabs and were removed from follow-up and 43 (8%) moved from the area or withdrew from the study) (Fig. 1, Supplementary Fig. 1). *S. aureus* grew from 3749 of 7009 post-recruitment swabs returned (53%) and was subsequently recovered from 73 (35%) individuals *S. aureus* negative at recruitment (recruitment-negatives), ten (5%) at the first swab after recruitment. All *S. aureus* were *spa*-typed; of the 297 *spa*-types observed, 197 (66%) were only seen in one individual. The 297 *spa*-types formed 157 groups with ≤2 differences, 82 were singletons and 22 could not be grouped because they were too short (Supplementary Table 2).

### Carrier index

**Figure 2:**
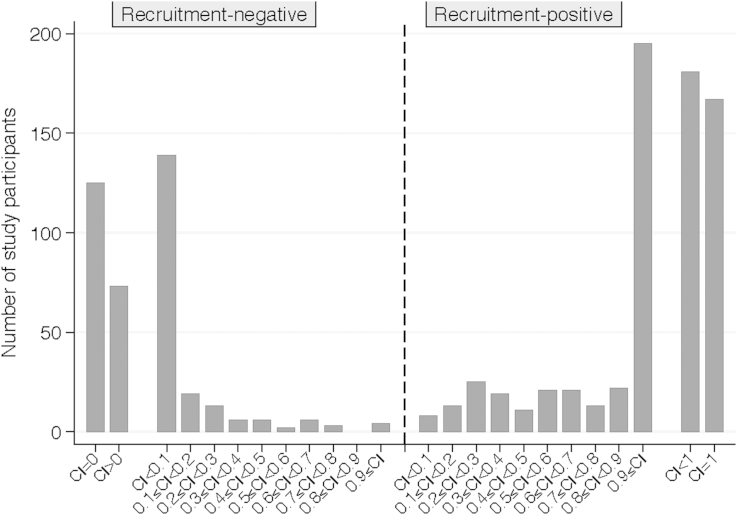
Carrier index.

**Figure 3:**
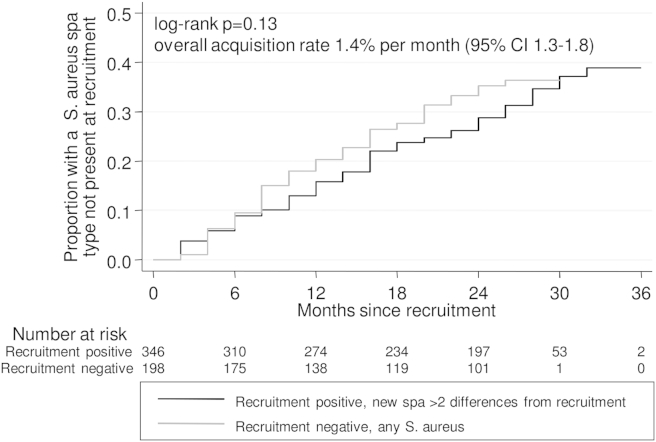
Months to acquisition of a new *S. aureus spa*-type in recruitment-positives and negatives. *Note*: not counting a *spa*-type first observed at the first post-recruitment swab as an acquisition, see Methods.

**Figure 4:**
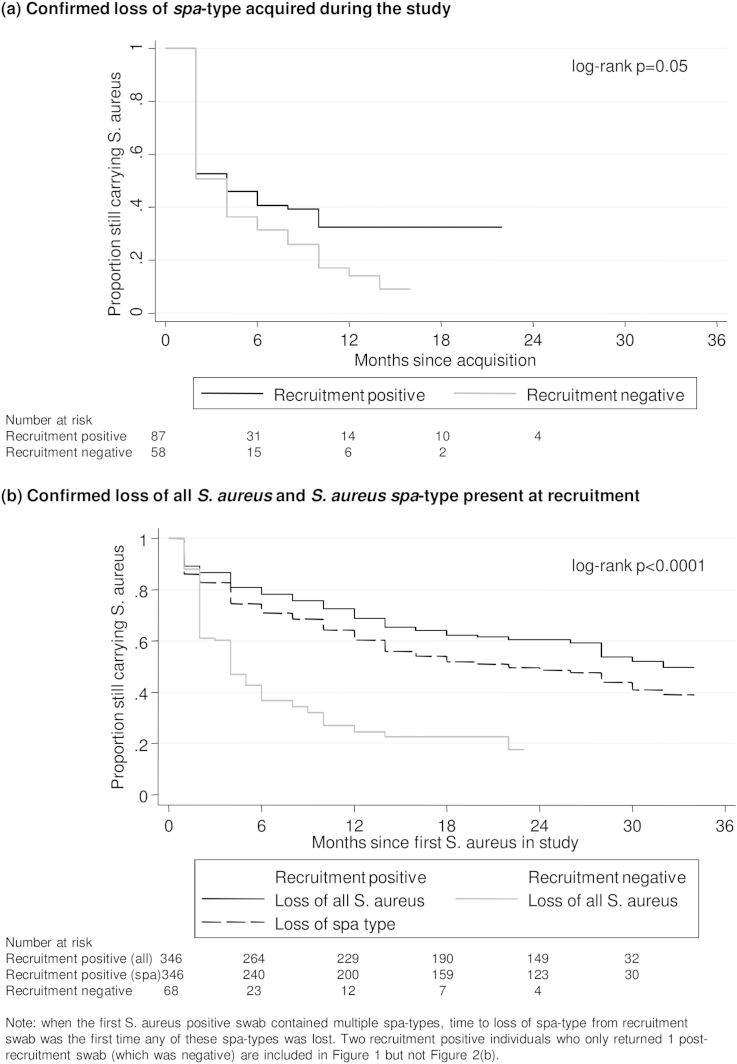
Months to confirmed loss of *S. aureus* in recruitment-positives and negatives. (a) Confirmed loss of *spa*-type acquired during the study. (b) Confirmed loss of all *S. aureus*, and of *S. aureus spa*-type present at recruitment in recruitment-positives. *Note*: when the first *S. aureus* positive swab contained multiple *spa*-types, time to loss of *spa*-type from recruitment swab was the first time any of these *spa*-types was lost.

Based on the carrier index (proportion of *S. aureus* positive swabs/swabs returned), just under half of the recruitment-positives carried *S. aureus* consistently throughout the study, and just over 60% of recruitment-negatives never carried *S. aureus* (Fig. 2). However, most of those with intermediate carrier indices had distinct phases of carriage of specific *spa*-types and phases of non-carriage. In particular, recruitment-positives lost carriage at similar rates throughout the study, leading to approximately equal numbers with carrier indices below one. We therefore estimated the time course over which recruitment-negatives became positive and recruitment-positives gained a new *spa*-type (“gain”, Fig. 3), and over which recruitment-positives became negative and recruitment-negatives who had become positive then lost carriage (“loss”, Fig. 4).

### *S. aureus* acquisition

162 (30%) of 544 participants returning ≥2 post-recruitment swabs acquired a new *spa*-type (with >2 differences) during follow-up, at a rate of 1.5% (95% CI 1.3–1.8%) per month. MRSA (EMRSA-15) was acquired by one individual. Similar percentages of recruitment-positives (29%) and recruitment-negatives (32%) acquired a new *spa*-type, and acquisition rates were similar (1.4% (95% CI 1.2–1.7%) and 1.8% (1.4–2.3%) per month respectively; log-rank *P* = 0.13, Fig. 3). There was no suggestion that acquisition rates plateaued over time (Fig. 3). Age was the strongest recruitment factor associated with rate of acquisition, which was faster in younger individuals (adjusted *P* = 0.01) (Table 1, Supplementary Table 2). Acquisition rates also varied independently with recruitment CC (global adjusted *P* = 0.04); being significantly faster in those with CC8 (adjusted *P* = 0.001), the CC including USA300, and with CC15 (adjusted *P* = 0.03).

In time-updated models including post-recruitment factors, having *S. aureus* isolated from the previous swab significantly decreased the rate of acquisition of a new *spa*-type (adjusted for Table 1 factors hazard ratio (a)HR = 0.61 (0.40–0.91), *P* = 0.02). Based on the analysis of recruitment factors above, we divided carriage of pre-existing *S. aureus* into CC8, CC15 or another CC, and found significant variation in this effect across these clonal complex groups (*P* = 0.002). Compared to those without pre-existing *S. aureus*, acquisition of a new *spa*-type occurred at similar rates in those with CC15 (aHR = 1.18 (0.60–2.31) and possibly at even higher rates in those with pre-existing CC8 (aHR = 2.03 (0.79–5.20); acquisition of a new *spa*-type was only reduced in those with other CCs (aHR = 0.50 (0.32–0.76)). Anti-staphylococcal antibiotics (see Supplementary Methods) were taken by 158/571 (28%) participants during the study; their use in the interval between the previous and current swab did not significantly affect *S. aureus* acquisition (aHR = 0.97 (0.49–1.91), *P* = 0.93). However, having received antibiotics more than two swabs ago increased the rate of *S. aureus* acquisition (aHR = 1.66 (1.16–2.38), *P* = 0.006), suggesting that individuals who lose *S. aureus* due to antibiotics are likely to re-acquire. There was no evidence that current inpatient admissions significantly affected *S. aureus* acquisition at the species or *spa-*level (adjusted *P* > 0.3) and the effects of previous antibiotics and co-colonisation remained when adjusted for one another, that is, were independent.

### *S. aureus* loss

We first considered loss of *S. aureus spa*-type in those in whom the date of acquisition was observed, that is those who acquired a new *spa*-type in the study and subsequently returned ≥2 swabs (*n* = 145; Fig. 4(a)). 98 (68%) subsequently lost this *spa*-type (53/87 (61%) recruitment-positives and 45/58 (78%) recruitment-negatives, log-rank *P* = 0.05). Median (IQR) carriage duration of acquired *spa*-types was two^2–10^ months in recruitment-negatives and two (2–>18) months in recruitment-positives. Loss rates varied substantially over time since acquisition (Supplementary Fig. 1(a)), averaging 19%/month (95% CI 15–24%) in the first four months versus 5%/month (3–8%) subsequently (3%/month (2–6%) in recruitment-positives versus 10%/month (5–18%) in recruitment-negatives) with no evidence of further slowing during the study.

We then considered loss of all *S. aureus* at the species level (Fig. 4(b)). 134 (39%) of 346 recruitment-positives returning ≥2 post-recruitment swabs subsequently lost all *S. aureus* during the study. Whilst overall loss rates were greater in recruitment-negatives subsequently observed to carry *S. aureus* (log-rank *P* < 0.0001), the difference in loss rates was largest early on (Supplementary Fig. 1(b)); after four months, losses occurred at low and more similar rates in both groups (2%/month (95% CI 1–2%) in recruitment-positives versus 6%/month (3–10%) in recruitment-negatives). First confirmed absence of any *spa*-type present at recruitment occurred at a slightly faster rate than loss of all *S. aureus* (Fig. 4(b)), indicating lost strains were often merely replaced. Age was independently associated with rate of *spa*-type loss, which was faster in younger individuals (adjusted *P* = 0.05; Table 1). More recent outpatient exposure, having more household members and being negative for *S. aureus* on recruitment were independent predictors of loss (adjusted *P* = 0.001, *P* = 0.03 and *P* < 0.0001 respectively). There was no evidence of an impact of recruitment CC on *spa*-type loss (adjusted global *P* = 0.42).

In time-updated models including post-recruitment factors, having multiple *spa*-types (differing by >2 repeats) in the previous swab had no significant effect on loss at the species level (adjusted for Table 1 factors aHR = 0.64 (95% CI 0.23–1.74), *P* = 0.38), but significantly increased loss of the original pre-existing *spa*-type (aHR = 3.40 (2.15–5.37), *P* < 0.001). Thus observations of multiple *spa*-types were commonly followed by replacement of the original with the new *spa*-type. Recent use of anti-staphylococcal antibiotics independently increased the rate of *S. aureus* loss at the species level (aHR = 2.51 (95% CI 1.54–4.10), *P* < 0.0001) (similar results for *spa*-type loss). There was no evidence that current inpatient admissions significantly affected *S. aureus* loss at the species or *spa*-level (adjusted *P* > 0.3).

### Exploration of long-term carriage patterns

(i) Long-term consistent carriage at the *S. aureus* species level

**Figure 5:**
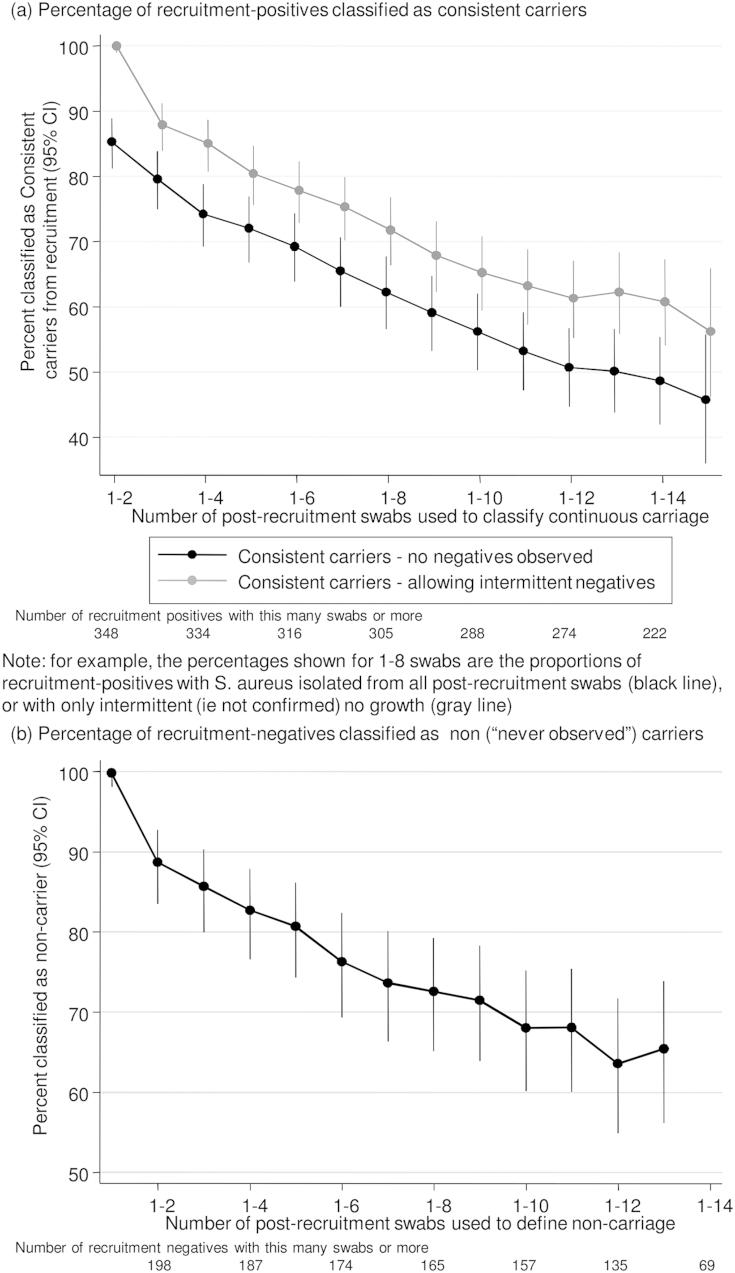
Impact of duration of follow-up on classification of carriage phenotypes. (a) Percentage of recruitment-positives classified as consistent carriers. *Note*: for example, the percentages shown for 1-8 swabs are the proportions of recruitment-positives with *S. aureus* isolated from all post-recruitment swabs (black line) or with only intermittent (i.e. not confirmed) no growth (grey line). (b) Percentage of recruitment-negatives classified as non (“never observed”) carriers.

To explore whether a consistent (long-term) carriage phenotype existed in our study, we combined the carrier index (Fig. 2) and time-to-loss (Fig. 4(b)) approaches to estimate the proportion of recruitment-positives observed to have carried *S. aureus* consistently in their first two, three, four, five etc swabs (Fig. 5(a)). The proportion of long-term consistent carriers declined linearly at least through to the first 12 swabs (∼24 months). After 12 swabs, confidence intervals were wide, and results were compatible with the ongoing low rates of loss seen in Supplementary Fig. 1. For example, of 140 individuals who were classified as consistent long-term carriers based on their first 12 swabs and who returned *≥*14 swabs, 11 (8%) subsequently lost carriage on two consecutive samples. Allowing single intermittent negative swabs increased estimates of consistent long-term carriers by ∼10%, but the relationship with number of swabs was similar (Fig. 5(a)). Of the 274 recruitment-positive participants returning ≥12 swabs, 157 (57%) never had two consecutive negative swabs, i.e. could be considered to have carried consistently long-term throughout the study. 4/61 (7%) recruitment-negatives returning ≥12 swabs with ≥1 positive could also be considered to have carried consistently long-term throughout the study (i.e. plausibly had an initial false-negative recruitment swab).

(ii) Long-term carriage at the *S. aureus spa*-type level

Of the 161 individuals without two consecutive negative swabs (i.e. defined long-term consistent carriers at the species level), 92 (57%) carried a single *spa*-type throughout without any other *spa*-type being observed, 45 (28%) carried a single *spa*-type throughout as well as gaining/losing other types; and 24 (15%) did not carry one *spa*-type consistently. Therefore 137/335 (41%) participants ever observed to carry *S. aureus* were consistent long-term carriers of the same *spa*-type, 135/274 (49%) recruitment-positives and 2/61 (3%) recruitment-negatives. Gaining/losing other *spa*-types was more common in persistent carriers of CC8 (3/3,100%) and CC15 (9/14,64%) than persistent carriers of other *spa*-types (33/120,28%) (*P* = 0.001), although numbers were small so results may not be robust.

(iii) “Non-carriage”

Taking a similar approach to explore a “never carriage” phenotype, the percentage of recruitment-negatives classified as non-carriers continued to decline linearly with increasing numbers of swabs. 90/151 (60%) recruitment-negatives returning ≥12 swabs never grew *S. aureus* during the study.

### Characteristics of different carriage phenotypes in those returning ≥12 swabs

The characteristics of those carrying one *spa*-type consistently long-term (allowing gain/loss of other *spa*-types), intermittent carriers of one or multiple *spa*-types and non-carriers are shown in Table 2 and Supplementary Table 4. Intermittent carriers had median (IQR) carrier index 0.33 (0.16–0.57) for their most commonly observed *spa*-type. Consistent carriers of one *spa*-type long-term appeared to differ in the CC of the *spa*-type they carried consistently, being more likely to carry CC22 (which includes EMRSA-15) (adjusted *P* = 0.03) and somewhat less likely to carry CC15 (*P* = 0.08) than intermittent carriers. Consistent carriers of one *spa*-type long-term were also less likely to have received anti-staphylococcal antibiotics, had fewer other household members and longer times since their last outpatient appointment (*P* = 0.04, 0.02 and 0.01 respectively).

## Discussion

In this large primary care-based study, we found 32% participants positive for *S. aureus* on a recruitment nasal swab, remarkably similar to *S. aureus* prevalence in other population studies, suggesting our results are likely generalisable.^1,2,11^ However, unlike the majority of other studies, our median follow-up of two years with bi-monthly swabs allowed detailed investigation of long-term carriage, and *spa*-typing every isolate enabled discrimination at the strain rather than the species level.

Our findings are compatible with a carriage spectrum in which the extremes are characterised by two phenotypes present at different proportions in recruitment-positives and negatives. The first is highly transient carriage, exemplified by most acquisitions in recruitment-negatives, who carried for a median of only two months. Interestingly the rate of acquisition of new *spa*-types was similar and approximately constant in recruitment-negatives and positives, and recruitment-positives lost newly acquired strains at only slightly slower rates than recruitment-negatives, suggesting that both are susceptible to highly transient carriage. The second carriage phenotype is long-term *S. aureus* carriage, exemplified by the much slower loss of recruitment *spa*-types in recruitment-positives, and low loss rates >4–6 months after acquisition in both recruitment-positives and negatives.

Our data could not fully support or refute the presence of a third “truly persistent” carriage phenotype as the proportion classified as consistent long-term carriers continued to decline as length of follow-up increased throughout the study. Further follow-up will be necessary to assess this definitively. Using our method of analysis, truly persistent carriage would be indicated by loss rates reducing to zero some time after 24–30 months (Supplementary Fig. 1(b)) with no further change in the proportion still carrying *S. aureus* (Figs. 4(b) and 5(a)). Other studies have defined “persistence” using more frequent sampling over shorter timescales,^11,12^ sometimes using quantitative culture^27^; when this study was set-up resource-constraints required a compromise between less intensive long-term versus more intensive short-term follow-up. One important study limitation is clearly the lack of a sampling point earlier than one month, e.g. at one week, which would have enabled us to investigate “persistent” carriers defined using van Nouwen's rule.^12^ The fact that these “persistent” carriers have been shown to differ significantly in characteristics such as clearance of a *S. aureus* inoculum,^19^ and host genetics,^13^ indicates that at least a subgroup form a distinct sub-population. However, we did have a sampling point at one month, and Fig. 5(a) demonstrates a clear ongoing linear decline in consistent long-term carriage even in those with two initial positive cultures, suggesting that a proportion with “persistent” carriage will not carry *S. aureus* long-term. In fact five of 17 “persistent” carriers (29%, 95% CI 10–56%) were not carrying *S. aureus* eight years later in the original study of Van den Bergh.^11^ Since van Nouwen et al. found that two qualitative and two qualitative + quantitative cultures had almost identical performance for classifying “persistence” in a validation set,^12^ it is unclear that doing quantitative cultures in our study would have materially altered this finding; we prioritised *spa*-typing all isolates over such quantitative culture.

Our findings suggest that “persistence” as previously defined^12,27^ could overestimate long-term carriage at the species level, and thus that there is no quick and reliable method to identify consistent long-term *S. aureus* carriers. Furthermore, 15% of long-term carriers at the species level in our study did not carry the same *spa*-type consistently (similarly to Ref.^28^). Whilst colonised with *S. aureus* long-term, the arrival of a new strain may still have the potential to alter outcomes, similarly to acquiring *S. aureus* de novo,^5^ highlighting the importance of using strain typing to identify truly persistent carriage. Assuming those followed long enough to identify this group were representative, “*spa*-consistent” long-term carriers would comprise 17% of those enrolled. Interestingly, two-thirds of these “*spa*-consistent” long-term carriers never had any other strain identified despite the long follow-up and the fact that multi-strain colonisation was actively investigated.

We found that the rate of new acquisitions increased linearly through the study (Fig. 3) and the proportion never observed to carry correspondingly decreased linearly (Fig. 5(b)). Our data are thus compatible with van Belkum's suggestion, based on experimental inoculation studies,^19^ that there are no true *S. aureus* non-carriers, i.e. that a fourth “never carriage” group does not exist. Whilst 90 participants returning ≥12 swabs never had *S. aureus* isolated from any study sample, the highly transient carriage that was observed suggests it could have been found at intermediate timepoints. Extrapolating from Fig. 3, 5–10 years follow-up would be needed to distinguish a never carriage phenotype (where the cumulative new acquisition probability would plateau) from continued acquisitions (where the cumulative new acquisition probability would reach 100%). The former scenario would imply that host, rather than bacterial, genetics determines this phenotype.

Despite this being the largest longitudinal study of *S. aureus* carriage to date, we failed to find strong predictors of gain, loss or persistence, possibly reflecting multifactorial causes and limited power to detect modest absolute differences of around 10%, given that the study was powered to detect 15% differences. Overall effects on loss, gain, and persistence were broadly compatible, although these reflect subtly different aspects of the underlying dynamics. Host effects likely reflected potential for *S. aureus* exposure (household members, students), underlying host-immunity (age, previous MSSA), and complex effects of health status (long-term illness, recent outpatient appointments). Interestingly receiving anti-staphylococcal antibiotics significantly increased the likelihood of losing *S. aureus* in the next swab, but also increased the likelihood of later acquisition. This is consistent with antibiotics only temporarily removing *S. aureus* from the nares, followed by re-acquisition from other body sites/close contacts, as in one study of artificial decolonisation and re-colonisation.^19^ These findings question the validity of *S. aureus* eradication as a concept, and suggest that reducing *S. aureus* load around high-risk procedures (e.g. through decontamination/prophylaxis pre-surgery) is a more biologically plausible approach to reducing *S. aureus* infection risk.

Unexpectedly, we found large effects of *spa*-type on acquisition and long-term consistent carriage. Compared to other CCs, more new *spa*-types were gained if CC8 or CC15 were present either at recruitment or in the previous swab, and CC15 in particular was less likely to be carried consistently long-term than intermittently, similar to findings based on using only two swabs to define “persistence”.^29^ Whilst pre-existing colonisation did not affect *S. aureus* loss at the species level, co-carriage was significantly associated with loss of the original strain. This demonstrates the highly dynamic nature of carried populations, and that nasal competition from particular strains is an important factor in co-carriage in vivo. Interestingly, CC8 includes USA300, so tolerance of co-carriage might contribute to the success of this lineage if it is also more readily acquired (not evaluable here due to low numbers of acquisitions of specific clones). Only CC22 was found in significantly more long-term carriers; this CC includes EMRSA-15, again potentially explaining this clone's success. This finding was implied by analyses of loss (Table 1). However, clearly the fact that the most widely dispersed CC8 and CC22 strains contain *mecA* is a confounding factor.

Our study had four main limitations. Firstly, participants were swabbed only in the nares, which likely missed some carriage even at the timepoints assayed^30,31^; a recent study suggests that this may have particularly underestimated carriage in younger people.^32^ However, swabbing of the nares enabled participant self-swabbing, which was vital for our study and was recently shown to have reasonable accuracy.^33^ Secondly, the bi-monthly sampling interval may have missed some transient carriage. However, it seems probable that those consistently observed to carry the same *spa*-type for >20 months (*n* = 137) would have also carried this *spa*-type at intermediate timepoints. Thirdly, smoking status was not available, but has been associated with reduced carriage in cross-sectional studies.^34–36^ Finally, since we combined unrelated CCs with less than ten isolates into a single group for analysis, and since numbers of isolates even in some larger CCs were still relatively modest, our findings on CCs should be confirmed in future studies. Our choice to perform overnight culture of swabs in enrichment medium only and not use direct plating may be seen both as a limitation and a strength. A limitation is that quantitation, which can predict persistent carriage,^12^ becomes impossible. A strength is that enrichment increases the sensitivity of the test and since resources prevented us from identifying isolates using both methods, we opted to maximise detection.

In conclusion, two years follow-up is insufficient to identify whether truly persistent long-term or “never” carriage phenotypes exist; this will require five-ten years follow-up (continuing in this study). Long-term *S. aureus* carriage at the *S. aureus* species level is not the same as long-term carriage at the *spa*-type level (observed in 57% and 49% of recruitment-positives respectively). Thus to identify long-term carriage reliably requires swabs over at least two years and *spa*-typing, including systematic methods for identifying co-colonisation, limiting the potential for accurate identification of long-term consistent carriage phenotypes for future genome-wide association studies. However, we have conclusively demonstrated bacterial lineage-specific effect on carriage dynamics. The transient carriage of *spa*-types with/without underlying persistent carriage, the lack of modifiable risk factors and the strong influence of antibiotics and strain-type on carriage acquisition, loss and persistence, highlights the dynamic nature of *S. aureus* as a human commensal. This emphasises the importance of focussing prevention efforts on reducing universal infection risk rather than eradication of carriage in individuals.^37,38^

## Funding

This work was supported by both the National Institute for Health Research (NIHR) under its Oxford Biomedical Research Centre Infection Theme, and the UKCRC Modernising Medical Microbiology Consortium, the latter being funded under the UKCRC Translational Infection Research Initiative supported by Medical Research Council, Biotechnology and Biological Sciences Research Council and the National Institute for Health Research on behalf of the Department of Health (Grant G0800778) and The Wellcome Trust (Grant 087646/Z/08/Z). DWC and TEAP are NIHR Senior Investigators. The views expressed in this publication are those of the author(s) and not necessarily those of the National Health Service, the NIHR or the Department of Health.

The funders had no role in study design, data collection, analysis, decision to publish, or manuscript preparation.

## Contributions

The study was conceived and designed by RM, DWC, TEAP, ASW, KK, RB and DM, with analysis performed by RM and ASW. HG, RF, RM and AV contributed to data acquisition. RM, ASW, KK, DM, TEAP and DCW contributed to data interpretation. RM wrote the first draft which all authors commented on, and all authors approved the final version. RM had full access to all the data in the study and takes responsibility for the integrity of the data and the accuracy of the data analysis; and the decision to submit for publication.

## Conflict of interest

No author has a conflict of interest.

## Figures and Tables

**Supplementary Figure 2:**
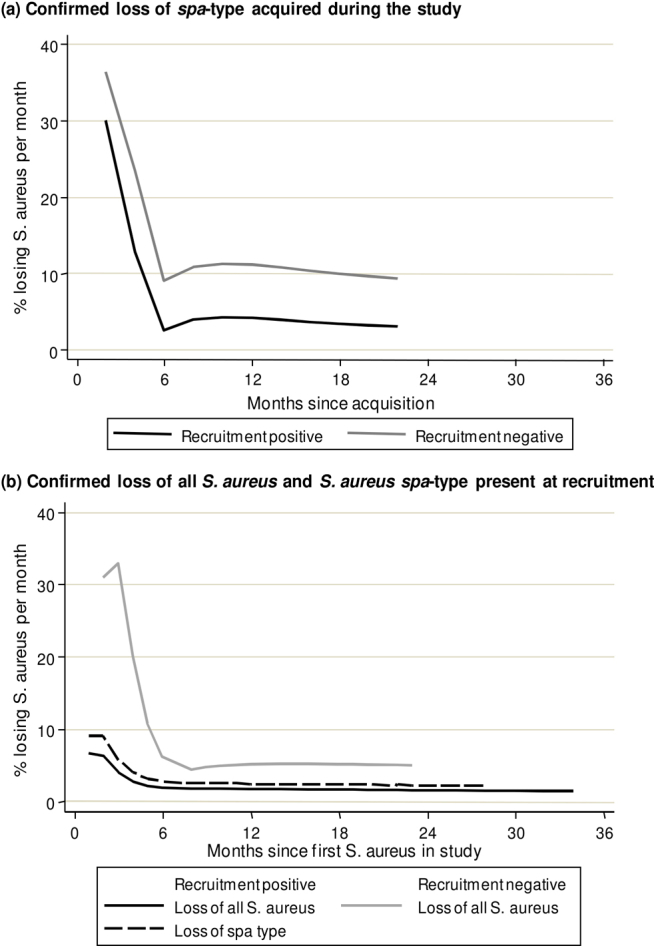
Rate of confirmed loss of *S. aureus* in recruitment-positives and negatives. (a) Confirmed loss of *spa*-type acquired during the study. (b) Confirmed loss of all *S. aureus* and *S. aureus spa*-type present at recruitment.

**Table 1:** Recruitment factors independently associated with acquisition of a new *spa*-type or confirmed loss of a *S. aureus spa*-type.

Factor (determined at recruitment)	*(effect in Cox model)*	Acquisition of a new *spa*-type (*N* = 523)			*P* value	Loss of *spa*-type (*N* = 401)			*P* value
		Acquire (*N* = 157) *n* (row %) or median (IQR)	Do not acquire (*N* = 366) *n* (row %) or median (IQR)	Hazard ratio (95% CI) from multivariable model		Number lose (*N* = 220) *n* (row %) or median (IQR)	Number do not lose (*N* = 181) *n* (row %) or median (IQR)	Hazard ratio (95% CI) from multivariable model	
***S*. *aureus* details (included by design)**									
Recruitment-negative	*No*	97 (29%)	242 (71%)	1.00		168 (50%)	169 (50%)	1.00	
	*Yes*	60 (33%)	124 (34%)	1.35 (0.82;2.22)	0.23	52 (81%)	12 (19%)	**3.23 (2.07;5.02)**	**<0.0001**
CC present at recruitment	*CC30 (spa-CC021) (reference category)*	23 (23%)	76 (77%)	1.00	**global 0.04**	45 (45%)	54 (55%)	1.00	global 0.42
	*CC15 (spa-CC084)*	16 (38%)	26 (62%)	**2.01 (1.06;3.83)**	**0.03**	22 (52%)	20 (48%)	1.31 (0.78;2.19)	0.31
	*spa-CC002*	6 (26%)	17 (74%)	1.04 (0.42;2.57)	0.94	12 (55%)	10 (45%)	1.45 (0.76;2.77)	0.26
	*CC22 (spa-CC005)*	7 (29%)	17 (71%)	1.40 (0.60;3.27)	0.44	7 (29%)	17 (71%)	0.57 (0.26;1.27)	0.17
	*spa-CC127*	6 (27%)	14 (73%)	1.36 (0.55;3.37)	0.51	13 (65%)	7 (35%)	1.46 (0.78;2.72)	0.24
	*spa-CC160*	3 (21%)	11 (79%)	1.07 (0.32;3.59)	0.91	7 (50%)	7 (50%)	1.18 (0.53;2.65)	0.68
	*CC8 (spa-CC024)*	7 (64%)	4 (36%)	**4.50 (1.91;10.6)**	**0.001**	7 (64%)	4 (36%)	1.28 (0.57;2.87)	0.55
	*Other CC*	29 (27%)	77 (73%)	1.34 (0.78;2.33)	0.29	55 (52%)	50 (48%)	1.36 (0.91;2.03)	0.13
**Participant characteristics (included by design)**									
Age (years)	*(Per 10 years older)*	40 (19;60)	54 (35;65)	**0.85 (0.75;0.97)**^b^	0.01	47 (22;62)	56 (37;67)	**0.90 (0.81;1.00)**^c^	**0.05**
Sex	*Female*	84 (30%)	192 (70%)	1.00		175 (68%)	83 (32%)	1.00	
	*Male*	73 (30%)	174 (70%)	1.13 (0.81;1.56)	0.47	98 (50%)	98 (50%)	0.94 (0.71;1.25)	0.68
Student	*No*	115 (27%)	318 (73%)	1.00		175 (52%)	163 (48%)	1.00	
	*Yes*	42 (47%)	48 (53%)	1.46 (0.86;2.47)	0.16	45 (71%)	18 (29%)	0.94 (0.58;1.52)	0.80
**Participant behaviour**									
Number of other household members	*Lives alone*	13 (19%)	54 (81%)	1.00		24 (44%)	31 (56%)	1.00	
	*1 household member*	55 (25%)	162 (75%)	*(trend, per category higher)*		74 (47%)	85 (53%)	*(trend, per category higher)*	
	*2/3 household members*	67 (37%)	116 (63%)	1.19 (0.95;1.51)	0.13	91 (62%)	55 (38%)	**1.22 (1.02;1.47)**	**0.03**
	*4 or more/shared*	22 (39%)	34 (61%)			31 (76%)	10 (34%)		
**Healthcare exposure**									
Days since last outpatient appointment^a^	*(Per year longer)*	322 (97;921)	299 (86;1106)	**0.95 (0.90;1.00)**	**0.06**	324 (106;900)	294 (84;1174)	**0.93 (0.89;0.97)**	**0.001**
**Co-morbidities**									
Has a long-term illness	*No*	83 (33%)	170 (67%)	1.00		110 (61%)	71 (39%)	1.00	
	*Yes*	74 (27%)	196 (73%)	0.91 (0.63;1.30)	0.59	110 (50%)	110 (50%)	**0.77 (0.57;1.04)**	**0.09**
Had MSSA previously	*No*	144 (29%)	350 (71%)	1.00		205 (54%)	173 (46%)	1.00	
	*Yes*	13 (45%)	16 (55%)	**1.68 (0.95;3.00)**	**0.08**	15 (65%)	8 (35%)	1.32 (0.77;2.26)	0.28

Note: IQR = inter-quartile range, CI = confidence interval. Including only individuals with general practice data at recruitment. See Supplementary Methods for details of model selection; factors with *P* < 0.10 shown in bold. Number of household contacts included as a trend term as point estimates increased monotonically in both adjusted models. All effects in the same direction as univariable models. See Supplementary Table 2 for univariable effects of these and other factors considered but not included.

a 6% of individuals with no outpatient appointment recorded set to 10 years, and all those with last outpatient appointment >10 years ago truncated at 10 years. Median (IQR) calculated in those with previous outpatient appointment.

b Effect stronger if number of household members not included in model: HR per 10 years older = 0.82 (95% CI 0.73–0.92) *P* = 0.001. Effects of other factors unchanged.

c Effect stronger if number of household members not included in model: HR per 10 years older = 0.86 (95% CI 0.78–0.94) *P* = 0.002. Effects of other factors unchanged.

**Table 2:** Impact of participant characteristics on long-term consistent carriage of a *S. aureus spa*-type versus intermittent carriage, and never observed carriage versus intermittent carriage, in participants returning ≥12 swabs (∼24 months).

Factor	*(effect in multinomial regression model)*	*S. aureus* long-term consistent *spa*-type carriers (*N* = 136): *n* (row %) or median (IQR)	*S. aureus* intermittent carriers (*N* = 192): *n* (row %) or median (IQR)	Long-term consistent *spa*-type versus intermittent carriage		Never observed carriers (*n* = 88):*n* (row%) or median (IQR)	Never observed versus intermittent carriage	
				RR (95% CI)	*P* value		RR (95% CI)	*P* value
Modal CC	*CC30 (spa-CC021) (reference category)*	43 (41%)	62 (59%)	1.00	global 0.13	**N/A**	**N/A**	**N/A**
	*CC15 (spa-CC084)*	14 (29%)	34 (71%)	0.49 (0.22;1.08)	**0.08**			
	*spa-CC002*	9 (33%)	18 (67%)	0.72 (0.28;1.88)	0.50			
	*CC22 (spa-CC005)*	16 (73%)	6 (27%)	**3.17 (1.09;9.24)**	**0.03**			
	*spa-CC127*	6 (33%)	12 (67%)	0.58 (0.19;1.75)	0.33			
	*spa-CC160*	5 (45%)	6 (54%)	0.96 (0.24;3.80)	0.95			
	*CC8 (spa-CC024)*	3 (30%)	7 (70%)	0.73 (0.16;3.35)	0.69			
	*Other CC (<10 people)*	40 (46%)	47 (54%)	1.06 (0.56;1.99)	0.85			
**Participant characteristics at recruitment**								
Age (years)	*(Per 10 years)*	60 (47;68)	50 (26;63)	1.05 (0.87;1.27)^b^	0.58	59 (41;65)	**1.27 (1.01;1.59)**^c^	**0.04**
Sex	*Female*	58 (27%)	109 (50%)	1.00		51 (23%)	1.00	
	*Male*	78 (39%)	83 (42%)	1.58 (0.96;2.58)	**0.07**	37 (19%)	0.83 (0.48;1.43)	0.50
Student	*No*	127 (36%)	155 (43%)	1.00		75 (21%)	1.00	
	*Yes*	9 (15%)	37 (63%)	0.41 (0.15;1.17)	**0.10**	13 (22%)	1.69 (0.60;4.78)	0.32
**Participant behaviour at recruitment**								
Number of other household members	*Lives alone*	25 (45%)	22 (40%)	1.00		8 (15%)	1.00	
	*1 household member*	71 (36%)	72 (37%)	*(trend, per category higher)*		53 (27%)	*(trend, per category higher)*	
	*2/3 household members*	36 (27%)	77 (57%)	0.64 (0.45;0.92)	**0.02**	21 (16%)	0.81 (0.54;1.22)	0.32
	*4 or more/shared*	4 (13%)	21 (67%)			6 (19%)		
**Participant behaviour at recruitment**								
Days since last outpatient appointment^a^	*(Per year longer)*	289 (83;1087)	324 (105;909)	1.11 (1.03;1.21)	**0.01**	241 (48;1123)	1.00 (0.91;1.10)	0.98
**Co-morbidities at recruitment**								
Has a long-term illness	*No*	46 (25%)	93 (50%)	1.00		48 (27%)	1.00	
	*Yes*	90 (39%)	99 (43%)	1.74 (0.98;3.10)	**0.06**	40 (17%)	0.58 (0.31;1.07)	**0.08**
Had MSSA previously	*No*	130 (33%)	179 (46%)	1.00		84 (21%)	1.00	
	*Yes*	6 (26%)	13 (57%)	0.62 (0.22;1.77)	0.37	4 (17%)	0.73 (0.23;2.36)	0.60
**Antibiotic exposure**								
Received anti-staphylococcal antibiotics during follow-up	*No*	90 (35%)	111 (44%)	1.00		54 (21%)	1.00	
	*Yes*	46 (29%)	81 (50%)	**0.58 (0.35;0.97)**	**0.04**	34 (21%)	0.87 (0.50;1.50)	0.61

Note: IQR = inter-quartile range; RR = risk ratio; CI = confidence interval. 9 individuals with missing general practice records excluded from analysis (1 persistent, 6 intermittent, 2 non-carriers). See Supplementary Methods for details of model selection; factors with *P* < 0.10 shown in bold. Number of household contacts included as a trend term. All effects in the same direction as univariable models. See Supplementary Table 3 for univariable effects of these and other factors considered but not included.

a 6% of individuals with no outpatient appointment recorded set to 10 years, and all those with last outpatient appointment >10 years ago truncated at 10 years. Median (IQR) calculated in those with previous outpatient appointment.

b Effect stronger if number of household members not included in model: HR per 10 years older = 1.16 (95% CI 0.98–1.38) *P* = 0.09. Effects of other factors unchanged.

c Effect stronger if number of household members not included in model: HR per 10 years older = 1.32 (95% CI 1.08–1.63) *P* = 0.007. Effects of other factors unchanged.
