# Supplementary material for: Dynamics of acquisition and loss of carriage of Staphylococcus aureus strains in the community: The effect of clonal complex
Source: J Infect. 2014 May;68(5):426–39. doi: 10.1016/j.jinf.2013.12.013 (PMC4003537; doi:10.1016/j.jinf.2013.12.013)
Supplement: Supplementary file 1 [file mmc1.docx]

**SUPPLEMENTARY MATERIAL**

Supplementary Methods

**Supplementary Table 1: Univariable impact of participant baseline characteristics and behaviour on *S. aureus* carriage at screening.**

**Supplementary Table 2: Univariable impact of previous healthcare exposure and co-morbidities on *S. aureus* carriage at screening.**

**Supplementary Table 3: Multivariable impact of recruitment risk factors on *S. aureus* positivity at recruitment**

**Supplementary Table 4: Frequency of every *spa*-type observed in the study.**

**Supplementary Table 5: Univariable impact of participant risk factors at recruitment on time to acquisition of a new *spa*-type or loss of a *S. aureus* *spa*-type. (a) Impact of recruitment characteristics and behaviour (b) previous healthcare exposure and co-morbidities.**

**Supplementary Table 6: Univariable impact of participant risk factors at recruitment on whether a *S. aureus spa*-type is carried long-term versus intermittently, or whether carriage is never observed versus intermittent. (a) Impact of recruitment characteristics and behaviour (b) previous healthcare exposure and co-morbidities.**

**Supplementary Table 7: Comparison of participants who were, and were not, followed up.**

Supplementary Figure 1: Flow diagram of participants followed up.

Supplementary Figure 2: Rate of confirmed loss of *S. aureus* in recruitment-positives and negatives

(a) Confirmed loss of *spa*-type acquired during the study

(b) Confirmed loss of all *S. aureus* and *S. aureus* *spa*-type present at recruitment

Footnote 1: Note: when the first *S. aureus* positive swab contained multiple *spa*-types, time to loss of *spa*-type from recruitment swab was the first time any of these *spa*-types was lost.

**SUPPLEMENTARY METHODS**

Epidemiological and healthcare information was collected from three sources. Participants completed a structured questionnaire at recruitment, including basic demographics; personal information about employment, household, and activities; and healthcare information not easily accessed in GP records (Supplementary Table 3a). Participants provided consent to access their GP records for healthcare risk factors, including appointments with GPs/nurses; hospital appointments outside OUH; and chronic healthcare conditions/history (Supplementary Table 3b). Where GP records were unavailable (9 (2%) and 16 (8%) recruitment-positives and negatives followed prospectively) participants were excluded from risk factor models; where details were missing (0-9%) risk factors were assumed absent. Participants also provided consent to access their OUH records for inpatient and outpatient visits. After two years follow-up, GP and OUH records were re-reviewed to collect information on antimicrobial use and inpatient episodes throughout follow-up. Anti-staphylococcal antibiotics were azithromycin, cefaclor, cefalexin, chlortetracycline, ciprofloxacin, clarithromycin, clindamycin, co-amoxyclav, doxycyclin, erythromycin, flucloxacillin, gentamicin, lymecycline, naseptin (chlorhexidine/neomycin), rifampicin, or tetracycline.

Cox regression was used to identify independent predictors of loss and acquisition; and logistic regression used to identify predictors of long-term carriage with the same *spa*-type versus other intermittent carriage (defining the modal CC as the most frequently observed CC per individual), and of never observed versus intermittent carriage. *S. aureus* CC was included in all multivariable models because this was the primary hypothesis, as were demographic factors reflecting the sampling design (age, gender, student). Backwards elimination on other factors (Supplementary Tables 3-4) used likelihood-based exit *P*=0.1 because many potential risk factors had univariable risk differences under the 15% on which the study was powered. Non-linearity in continuous factors was assessed using fractional polynomials (Stata *mfp* command, non-linearity criterion *P*=0.01) [1]. As each of the four outcomes reflects the underlying dynamics of the carriage state, final models included all factors identified as an independent predictor of any outcome (Tables 1-2).

SUPPLEMENTARY REFERENCES

1. Royston P, Altman DG. Regression Using Fractional Polynomials of Continuous Covariates - Parsimonious Parametric Modeling. Appl Stat-J Roy St C. 1994; **43**(3): 429-67.

**Supplementary Table 1: Univariable impact of participant baseline characteristics and behaviour on *S. aureus* carriage at screening.**

|  | | **Effect in logistic regression** | ***S. aureus* carriage at baseline (n=360) N (%) or median (IQR)†** | **No carriage (n=763) N (%) or median (IQR)†** | **Univariable *P* value carriage versus no carriage** | **Univariable OR**  **(95% CI)** | |
| --- | --- | --- | --- | --- | --- | --- | --- |
| Participant characteristics: | | | | | | | |
| Age | Per 10 years older | | 53 (33;65) | 56 (39;68) | 0·049 | 0·88 (0·99;1·00) | |
| Sex | Male | | 184 (51%) | 299 (39%) | <0·001 | 1·62 (1·26;2·09) | |
| Student | Yes (vs no) | | 39 (11%) | 82 (11%) | 0·97 | 1·01 (0·67;1·51) | |
| Ethnic background | White British/Irish | | 327 (91%) | 690 (90%) | 0·88 | 1·00 | |
|  | Other white | | 21 (6%) | 43 (6%) |  | 1·03 (0·60;1·77) | |
|  | Other ethnicity | | 12 (3%) | 30 (4%) |  | 0·84 (0·43;1·67) | |
| Participant behaviour | | | | | | | |
| Current employment | | Yes (vs no) | 190 (53%) | 330 (43%) | 0·003 | 1·47 (1·14;1·89) | |
| Healthcare related employment | | Yes (vs no) | 76 (21%) | 181 (24%) | 0·33 | 0·86 (0·64;1·17) | |
| Number of other household members | | Lives alone | 59 (16%) | 117 (15%) | 0·46 | 1·00 | |
|  |  | 1 household member | 142 (39%) | 343 (45%) |  | 0·82 (0·57;1·19) | |
|  |  | 2/3 household members | 123 (34%) | 227 (30%) |  | 1·07 (0·73;1·57) | |
|  |  | 4 or more | 31 (9%) | 63 (8%) |  | 0·98 (0·57; 1·66) | |
|  |  | Shared accommodation | 5 (1%) | 13 (2%) |  | 0·76 (0·26;2·24) | |
| Number of other household members with healthcare contact | | Zero | 297 (83%) | 604 (79%) | 0·16 | 1·0 |  |
|  |  | One | 57 (16%) | 152 (20%) |  | 0·76 (0·55;1·07) |  |
|  |  | Two or more | 6 (2%) | 7 (1%) |  | 1·74 (0·58;5·23) |  |
| Current participation in contact sport | | Yes (vs no) | 135 (38%) | 237 (31%) | 0·03 | 1·33 (1·02;1·73) |  |
| Looks after anyone with a disability/old age | | Yes (vs no) | 65 (18%) | 116 (15%) | 0·23 | 1·23 (0·88;1·72) |  |

Note: IQR=Inter-quartile range; OR=Odds Ratio; CI=Confidence Interval

**Supplementary Table 2: Univariable impact of previous healthcare exposure and co-morbidities on *S. aureus* carriage at screening.**

|  | **Effect in logistic regression** | ***S. aureus* carriage at baseline (n=351) N (%) or median (IQR)†** | **No carriage (n=491) N (%) or median (IQR)†** | **Univariable *P* value carriage versus no carriage** | **Univariable OR (95% CI)** |
| --- | --- | --- | --- | --- | --- |
| Previous healthcare exposure | | | | | |
| Ever been an in-patient visit  *Unknown* | Yes (vs no) | 295 (84%)  *0 (0%)* | 391 (80%)  *1 (0*·*2%)* | 0·11 | 1·35 (0·94;1·93) |
| Days since in patient episode | Per 30 days | 986 (346;2853) | 1044 (288;3092) | 0·89 | 1·00 (1·00;1·00) |
| Ever been an out-patient  *Unknown* | Yes (vs no) | 330 (94%)  *0 (0%)* | 469 (96%)  *2 (0*·*4%)* | 0·33 | 0·73 (0·40;1·36) |
| Out-patient appointment | No | 21 (6%) | 22 (4%) | 0·10 |  |
|  | Within 30 days | 37 (11%) | 75 (15%) |  | 0·52 (0·25;1·06) |
|  | > 30 days ago | 293 (83%) | 394 (80%) |  | 0·78 (0·42;1·44) |
| Ever had a GP appointment  *Unknown* | Yes (vs no) | 348 (100%)  *0 (0%)* | 490 (100%)  *0 (0%)* | 0·21 | 0·23 (0·02;2·29) |
| Days since GP appointment | Per 30 days | 58 (22;152) | 72 (25;182) | 0·63 | 1·00 (0·99;1·02) |
| Ever had a practice nurse appointment  *Unknown* | Yes (vs no) | 341 (97%)  *2 (0*·*4%)* | 476 (97%)  *1 (0*·*3%)* | 0·86 | 1·07 (0·47;2·42) |
| Days since practice nurse appointment | Per 30 days | 109 (34;320) | 108 (29;311) | 0·41 | 1·00 (0·99;1·01) |
| Ever had a district nurse appointment  *Unknown* | Yes (vs no) | 63 (18%)  *5 (1%)* | 72 (15%)  *11 (2%)* | 0·20 | 1·27 (0·88;1·84) |
| Days since district nurse appointment | Per 30 days | 2544 (679;5661) | 1697 (265;4821) | 0·08 | 1·01 (1·00;1·01)) |
| Co-morbidities | | | | | |
| Has a long-term illness  *Unknown* | Yes (vs no) | 203 (58%)  *0 (0%)* | 261 (53%)  *1 (0*·*2%)* | 0·19 | 1·20 (0·91;1·59) |
| Ever had chemotherapy  *Unknown* | Yes (vs no) | 7 (2%)  *4 (1%)* | 21 (4%)  *2 (0*·*4%)* | 0·08 | 0·46 (0·19;1·08) |
| Ever had renal dialysis  *Unknown* | Yes (vs no) | 3 (1%)  *3 (1%)* | 1 (0·2%)  *2 (0*·*4%)* | 0·21 | 4·22 (0·44;40·8) |
| Ever had surgery  *Unknown* | Yes (vs no) | 267 (76%)  *2 (1%)* | 364 (74%)  *0 (0%)* | 0·52 | 1·11 (0·81;1·52) |
| Days since surgery | Per 30 days | 2575 (824;6180) | 1965 (549;5170) | 0·05 | 1·00 (1·00;1·01) |
| Ever prescribed oral steroids  *Unknown* | Yes (vs no) | 52 (15%)  *7 (3%)* | 82 (17%)  *15 (3%)* | 0·46 | 0·87 (0·59;1·27) |
| Treatment for skin conditions in last 30 days  *Unknown* | Yes (vs no) | 50 (14%)  *5 (2%)* | 47 (10%)  *4 (1%)* | 0·04 | 1·57 (1·03;2·40) |
| Ever had vascular access  *Unknown* | Yes (vs no) | 237 (67%)  *16 (5%)* | 314 (64%)  *21 (4%)* | 0·50 | 1720 (0·88;1·57) |
| Ever had a catheter  *Unknown* | Yes (vs no) | 79 (23%)  *18 (5%)* | 129 (27%)  *25 (5%)* | 0·21 | 0·82 (0·59;1·12) |
| Ever prescribed antibiotics  *Unknown* | Yes (vs no) | 333 (95%)  *5 (1%)* | 474 (97%)  *7 (1%)* | 0·34 | 0·51 (0·77;2·96) |
| Days since antibiotics | Per 30 days | 549 (183;1889) | 685 (174;2085) | 0·24 | 1·00 (1·00;1·00) |
| Had MRSA previously  *Unknown* | Yes (vs no) | 4 (1%)  *9 (3%)* | 3 (1%)  *18 (4%)* | 0·48 | 1·88 (0·42;8·43) |
| Had MSSA previously  *Unknown* | Yes (vs no) | 20 (6%)  *9 (3%)* | 27 (6%)  *18 (4%)* | 0·90 | 1·04 (0·57; 1·88) |

Note: IQR=Inter-quartile range; OR=Odds ratio. Days since last out-patient appointment converted into categorical variable as relationship non-linear. Missing values assumed as no, as risk factors likely to be recorded in patient records.

**Supplementary Table 3: Multivariable impact of recruitment risk factors on *S. aureus* positivity at recruitment**

| Baseline Factor | *(effect in logistic regression)* | *S. aureus* positive: N (%) or median (IQR) | *S. aureus* negative: N (%) or median (IQR) | **Multivariable model (i)** | | **Multivariable model (ii)** | |
| --- | --- | --- | --- | --- | --- | --- | --- |
|  |  |  |  | OR (95% CI) | *P* value | OR (95% CI) | *P* value |
| Personal details questionnaire completed | | N=360 (100%) | N=763 (100%) | N=1123 |  | N=842 |  |
| Participant characteristics | |  |  |  |  |  |  |
| Age (years) | *(per 10 years older)* | 53 (33;65) | 56 (39;68) | 0·96 (0·89;1·03) | 0·22 | 0·99 (0·91;1·08) | 0·89 |
| Sex | Male (vs female) | 184 (51%) | 299 (39%) | 1·67 (1·29;2·15) | <0·0001 | 1·67 (1·05;2·23) | <0·0001 |
| Participant behaviour | |  |  |  |  |  |  |
| Current employment | Yes (vs no) | 190 (53%) | 330 (43%) | 1·48 (1·01;1·90) | 0·003 | 1·40 (1·05;1·86) | 0·02 |
| Number of other household members with healthcare contact | 0 | 297 (83%) | 604 (79%) | 1·00 | 0·27 | 1·00 | 0·75 |
|  | 1 | 57 (16%) | 152 (20%) | 0·79 (0·56;1·11) |  | 0·87 (0·60;1·27) |  |
|  | 2 or more | 6 (2%) | 6 (1%) | 1·62 (0·53;4·95) |  | 1·10 (0·33;3·72) |  |
| Current participation in contact sport | Yes (vs no) | 135 (38%) | 237 (31%) | 1·34 (1·02;1·75) | 0·03 | 1·28 (0·95;1·73) | 0·10 |
| GP record information available | | N=351 (98%) | N=491 (64%) |  |  |  |  |
| Previous healthcare exposure | |  |  |  |  |  |  |
| Ever been an inpatient | Yes (vs no) | 295 (84%) | 391 (80%) |  |  | 1·53 (1·04;2·30) | 0·03 |
| Outpatient appointment | No | 21 (6%) | 22 (4%) |  |  | 1·00 | 0·13 |
|  | Within last 30 days | 37 (11%) | 75 (15%) |  |  | 0·50 (0·23;1·07) |  |
|  | >30 days ago | 293 (83%) | 394 (80%) |  |  | 0·74 (0·38;1·42) |  |
| District nurse appointment | No | 288 (82%) | 419 (85%) |  |  | 1·00 | 0·04 |
|  | Within last 30 days | 1 (0·3%) | 5 (1%) |  |  | 0·27 (0·03;2·46) |  |
|  | >30 days ago | 62 (18%) | 62 (13%) |  |  | 1·56 (1·04;2·30) |  |
| Co-morbidities | |  |  |  |  |  |  |
| Ever had chemotherapy | Yes (vs no) | 7 (2%) | 21 (4%) |  |  | 0·46 (0·19;1·12) | 0·08 |
| Surgery | No | 84 (24%) | 127 (26%) |  |  | 1 | 0·28 |
|  | Within last 30 days | 7 (0·6%) | 13 (3%) |  |  | 0·44 (0·15;1·32) |  |
|  | >30 days ago | 260 (74%) | 351 (71%) |  |  | 1·02 (0·68;1·08) |  |
| Treatment for skin conditions in last 30 days | Yes (vs no) | 50 (14%) | 47 (10%) |  |  | 1·62 (1·04;2·52) | 0·03 |

**Note: IQR=Inter-quartile range; OR=odds ratio; CI=Confidence Interval. Multivariable model (i) included all factors univariably *P*<0**·**2 from the personal details questionnaire (complete data); model (ii) was restricted to those with GP information available (842/1123 (75%)) and also included factors univariably *P*<0**·**2 from GP (and OUH) records, excluding has a long-term illness which was collinear with other factors due to overlapping definitions. All multivariable effects shown are in the same direction as univariable effects. A small number of patients (1-43(1-9%)) for whom particular details of previous healthcare exposure could not be identified from GP records were treated as not having exposure. Personal details with *P*>0**·**2 in univariable models were: whether participant was a student; ethnic background; healthcare related employment and number of other household members; looks after anyone with a disability/old age. GP record *P*>0**·**2 in univariable models: days since last inpatient episode; ever had a GP appointment before recruitment; days since last GP appointment; ever had a practice nurse appointment; days since last practice nurse appointment; ever had renal dialysis; ever prescribed oral steroids; ever had vascular access; ever had a catheter; ever prescribed antibiotics; days since last antibiotics; previous MRSA or MSSA (any site).**

**Supplementary Table 4: Frequency of every *spa*-type observed in the study.**

| *spa* group | *spa*-type | *spa* CC | Equivalent MLST CC (where used) | Repeat pattern | Number of participants | Number of swabs from recruitment-positives | Number of swabs from recruitment-negatives | Number of swabs with MRSA |
| --- | --- | --- | --- | --- | --- | --- | --- | --- |
| 2 | t105 | CC2 |  | 26-23-17-34-17-20-17-17-16 | 1 | 3 | 0 | 0 |
| 2 | t548 | CC2 |  | 26-23-17-34-17-20-17-12-16 | 1 | 5 | 0 | 0 |
| 2 | t2115 | CC2 |  | 26-23-17-13-17-20-17-17-16 | 1 | 3 | 0 | 0 |
| 2 | t7032 | CC2 |  | 26-23-17-308-17-20-17-12-16 | 1 | 0 | 1 | 0 |
| 2 | t053 | CC2 |  | 26-23-17-34-17-20-17-12-17-34 | 1 | 1 | 0 | 0 |
| 2 | t002 | CC2 |  | 26-23-17-34-17-20-17-12-17-16 | 22 | 89 | 17 | 0 |
| 2 | t071 | CC2 |  | 26-23-23-17-34-17-20-17-12-17-16 | 1 | 7 | 0 | 0 |
| 2 | t088 | CC2 |  | 26-23-17-34-17-20-17-12-12-17-16 | 1 | 1 | 0 | 0 |
| 2 | t570 | CC2 |  | 07-23-17-34-17-20-17-12-17-16 | 1 | 3 | 0 | 0 |
| 2 | t8084 | CC2 |  | 26-23-133-34-17-20-17-12-17-16 | 1 | 3 | 0 | 0 |
| 5 | t005 | CC5 | CC22 | 26-23-13-23-31-05-17-25-17-25-16-28 | 11 | 106 | 0 | 0 |
| 5 | t006 | CC5 | CC22 | 26-23-13-23-31-05-17-17-25-16-28 | 1 | 13 | 0 | 0 |
| 5 | t022 | CC5 | CC22 | 26-23-13-23-31-29-17-31-29-17-25-17-25-16-28 | 1 | 4 | 0 | 0 |
| 5 | t032 | CC5 | CC22 | 26-23-23-13-23-31-29-17-31-29-17-25-17-25-16-28 | 6 | 43 | 0 | 35 |
| 5 | t192 | CC5 | CC22 | 26-23-13-23-31-05-17-25-17-25-16-16-28 | 2 | 19 | 0 | 0 |
| 5 | t379 | CC5 | CC22 | 26-23-23-13-23-31-29-17-25-17-25-16-28 | 2 | 32 | 0 | 14 |
| 5 | t449 | CC5 | CC22 | 26-23-13-23-31-05-05-17-25-17-25-16-28 | 1 | 10 | 0 | 0 |
| 5 | t910 | CC5 | CC22 | 26-23-23-13-23-31-29-17-25-16-28 | 1 | 1 | 0 | 1 |
| 5 | t1036 | CC5 | CC22 | 26-23-23-13-17-31-29-17-25-17-25-16-28 | 1 | 12 | 0 | 12 |
| 5 | t6703 | CC5 | CC22 | 26-23-13-23-31-05-17-31-17-25-16-28 | 1 | 16 | 0 | 0 |
| 5 | t6813 | CC32 |  | 399-23-23-13-23-31-29-17-31-29-17-25-17-25-16-28 | 1 | 1 | 0 | 1 |
| 5 | t6799 | CC5 | CC22 | 26-23-13-23-31-05-05-17-25-17-25-16-16-28 | 1 | 18 | 0 | 0 |
| 5 | t6831 | CC5 | CC22 | 26-23-13-13-23-31-05-17-25-17-25-16-28 | 1 | 0 | 1 | 0 |
| 5 | t7421 | CC5 | CC22 | 26-114-13-23-31-05-17-25-16-28 | 1 | 1 | 0 | 0 |
| 8 | t008 | CC24 | CC8 | 11-19-12-21-17-34-24-34-22-25 | 16 | 33 | 5 | 0 |
| 8 | t068 | CC24 | CC8 | 11-19-19-12-21-17-34-24-34-22-25 | 1 | 1 | 0 | 0 |
| 8 | t1476 | CC24 | CC8 | 11-10-17-34-24-34-22-25 | 1 | 1 | 0 | 0 |
| 8 | t2849 | CC24 | CC8 | 11-19-17-34-24-34-22-25 | 1 | 6 | 0 | 0 |
| 15 | t015 | CC21 | CC30 | 08-16-02-16-34-13-17-34-16-34 | 11 | 41 | 3 | 0 |
| 15 | t050 | CC21 | CC30 | 08-16-02-16-34-34-17-34-16-34 | 4 | 9 | 1 | 0 |
| 15 | t589 | CC21 | CC30 | 08-16-02-16-34-34-13-17-34-16-34 | 1 | 2 | 0 | 0 |
| 15 | t630 | CC21 | CC30 | 08-16-02-16-34-17-34-16-34 | 3 | 14 | 1 | 0 |
| 15 | t908 | CC21 | CC30 | 08-16-02-16-34-13-17-34-34-16-34 | 1 | 4 | 0 | 0 |
| 15 | t6793 | CC21 | CC30 | 08-16-02-16-34-13-17-397-16-34 | 1 | 3 | 0 | 0 |
| 24 | t024 | CC24 | CC8 | 11-12-21-17-34-24-34-22-25 | 7 | 25 | 1 | 0 |
| 24 | t701 | CC24 | CC8 | 11-10-21-17-34-24-34-22-25-25 | 1 | 0 | 1 | 0 |
| 24 | t2455 | CC24 | CC8 | 11-12-21-21-17-34-24-34-22-25 | 1 | 12 | 0 | 0 |
| 24 | t9572 | CC24 | CC8 | 11-12-21-21-21-17-34-24-34-22-25 | 1 | 1 | 0 | 0 |
| 78 | t056 | CC078/349 |  | 04-20-12-17-20-17-12-17-17 | 2 | 15 | 6 | 0 |
| 78 | t078 | CC078/349 |  | 04-21-12-41-20-17-12-12-17 | 6 | 38 | 0 | 0 |
| 78 | t258 | CC078/349 |  | 04-21-12-41-20-17-12-12-12-17 | 2 | 8 | 0 | 0 |
| 78 | t349 | CC078/349 |  | 04-21-12-17-20-17-12-12-17 | 2 | 4 | 0 | 0 |
| 78 | t401 | CC078/349 |  | 04-21-12-17-20-17-12-17 | 1 | 15 | 0 | 0 |
| 78 | t1521 | CC078/349 |  | 04-21-12-41-20-17-12-12 | 1 | 4 | 0 | 0 |
| 84 | t084 | CC84 | CC15 | 07-23-12-34-34-12-12-23-02-12-23 | 27 | 128 | 8 | 0 |
| 84 | t085 | CC84 | CC15 | 07-23-12-34-34-12-23-02-12-23 | 6 | 36 | 1 | 0 |
| 84 | t120 | CC84 | CC15 | 07-23-12-12-34-34-12-12-23-02-12-23 | 1 | 2 | 0 | 0 |
| 84 | t144 | CC84 | CC15 | 07-23-12-34-34-12-12-23-02-02-12-23 | 1 | 1 | 0 | 0 |
| 84 | t279 | CC84 | CC15 | 07-23-12-34-34-34-12-12-23-02-12-23 | 1 | 3 | 0 | 0 |
| 84 | t346 | CC84 | CC15 | 07-23-12-34-12-12-23-02-12-23 | 6 | 33 | 2 | 0 |
| 84 | t360 | CC84 | CC15 | 07-23-12-34-12-23-02-12-23 | 4 | 29 | 1 | 0 |
| 84 | t491 | CC84 | CC15 | 26-23-12-34-34-12-12-23-02-12-23 | 2 | 2 | 1 | 0 |
| 84 | t499 | CC84 | CC15 | 07-23-12-12-34-12-12-23-02-12-23 | 1 | 7 | 0 | 0 |
| 84 | t774 | CC84 | CC15 | 07-23-12-34-34-12-12-12-23-02-12-23 | 2 | 6 | 0 | 0 |
| 84 | t1492 | CC84 | CC15 | 07-23-12-34-34-34-12-23-02-12-23 | 1 | 6 | 0 | 0 |
| 84 | t1716 | CC84 | CC15 | 07-23-12-12-12-23-02-12-23 | 1 | 13 | 0 | 0 |
| 84 | t2074 | CC84 | CC15 | 07-23-12-12-12-12-23-02-12-23 | 1 | 5 | 0 | 0 |
| 84 | t6817 | CC84 | CC15 | 07-23-12-34-34-12-12-12-23-23-02-12-23 | 2 | 5 | 0 | 0 |
| 84 | t7031 | CC84 | CC15 | 07-23-12-34-34-12-12-23-02-12-34-34-12-12-23-02-12-23 | 2 | 2 | 3 | 0 |
| 84 | t9162 | CC84 | CC15 | 07-23-12-12-34-12-12-20-02-12-23 | 1 | 1 | 0 | 0 |
| 89 | t089 | CC89 |  | 04-33-31-12-16-34-16-12-33-34 | 8 | 70 | 1 | 0 |
| 89 | t5753 | CC89 |  | 04-33-31-12-16-34-16-16-12-33-34 | 1 | 1 | 0 | 0 |
| 89 | t6798 | CC89 |  | 04-33-31-12-16-34-34-16-12-33-34 | 1 | 1 | 0 | 0 |
| 91 | t091 | CC84 | CC15 | 07-23-21-17-34-12-23-02-12-23 | 15 | 48 | 17 | 0 |
| 91 | t1685 | CC84 | CC15 | 07-23-21-17-34-12-23-02-02-12-23 | 1 | 10 | 0 | 0 |
| 94 | t335 | CC84 | CC15 | 07-23-12-34-34-12-23 | 1 | 7 | 0 | 0 |
| 94 | t434 | CC84 | CC15 | 07-23-12-34-34 | 1 | 1 | 0 | 0 |
| 94 | t1877 | CC84 | CC15 | 07-23-12-34-12-12-23 | 1 | 4 | 0 | 0 |
| 127 | t127 | CC127 |  | 07-23-21-16-34-33-13 | 14 | 104 | 6 | 0 |
| 127 | t177 | CC127 |  | 26-23-21-16-34-33-13 | 1 | 11 | 0 | 0 |
| 127 | t948 | CC127 |  | 07-23-21-16-13-33-13 | 1 | 2 | 0 | 0 |
| 136 | t136 | CC136 |  | 04-44-33-31-12-16-34-16-12-25-22-22-34 | 6 | 45 | 1 | 0 |
| 136 | t166 | CC136 |  | 04-44-33-31-12-16-34-16-12-25-22-34 | 4 | 37 | 0 | 0 |
| 136 | t6415 | CC136 |  | 04-44-33-31-12-16-16-34-16-12-25-22-22-34 | 2 | 14 | 1 | 0 |
| 159 | t159 | CC659 |  | 14-44-13-12-17-17-23-18-17 | 1 | 1 | 0 | 0 |
| 159 | t645 | CC659 |  | 14-44-13-12-17-23-18-17 | 2 | 13 | 0 | 0 |
| 160 | t160 | CC160 |  | 07-23-21-24-33-22-17 | 14 | 64 | 1 | 0 |
| 160 | t2098 | CC160 |  | 07-23-21-24-33-21-17 | 1 | 4 | 0 | 0 |
| 164 | t164 | CC164 |  | 07-06-17-21-34-34-22-34 | 7 | 70 | 3 | 0 |
| 164 | t731 | CC164 |  | 07-06-17-21-34-22-34 | 1 | 2 | 0 | 0 |
| 164 | t996 | CC164 |  | 07-06-17-21-34-34-34-22-34 | 1 | 9 | 0 | 0 |
| 189 | t189 | CC127 |  | 07-23-12-21-17-34 | 8 | 50 | 1 | 0 |
| 216 | t216 | CC216 |  | 04-20-17-20-17-31-16-34 | 13 | 41 | 16 | 0 |
| 216 | t316 | CC216 |  | 04-20-17-31-16-34 | 2 | 2 | 2 | 0 |
| 216 | t471 | CC216 |  | 04-02-17-20-17-31-16-34 | 1 | 4 | 0 | 0 |
| 216 | t2930 | CC216 |  | 04-20-17-20-17-20-17-31-16-34 | 1 | 14 | 0 | 0 |
| 223 | t223 | CC5 | CC22 | 26-23-13-23-05-17-25-17-25-16-28 | 2 | 13 | 0 | 0 |
| 223 | t3304 | CC5 | CC22 | 26-23-20-31-05-17-25-17-25-16-28 | 1 | 15 | 0 | 0 |
| 223 | t6942 | CC5 | CC22 | 26-23-20-31-05-17-25-16-16-28 | 1 | 1 | 0 | 0 |
| 223 | t7049 | CC5 | CC22 | 26-23-20-31-05-17-25-17-25-28 | 1 | 1 | 0 | 0 |
| 228 | t228 | CC84 | CC15 | 14-12-34-34-12-12-23-02-12-23 | 2 | 18 | 0 | 0 |
| 228 | t1885 | CC84 | CC15 | 14-12-34-12-12-23-02-12-23 | 2 | 7 | 0 | 0 |
| 228 | t2556 | CC84 | CC15 | 14-12-34-34-12-23-02-12-23 | 1 | 0 | 2 | 0 |
| 228 | t3097 | CC84 | CC15 | 14-12-34-34-34-12-12-23-02-12-23 | 1 | 2 | 0 | 0 |
| 230 | t230 | CC21 | CC30 | 08-16-02-16-34 | 7 | 38 | 11 | 0 |
| 230 | t550 | CC21 | CC30 | 08-17-34-16-34 | 1 | 10 | 0 | 0 |
| 321 | t321 | CC127 |  | 07-23-16-34-33-13 | 1 | 4 | 0 | 0 |
| 321 | t1908 | CC127 |  | 07-22-16-34-33-13 | 1 | 3 | 0 | 0 |
| 359 | t267 | CC359/938 |  | 07-23-12-21-17-34-34-34-33-34 | 4 | 3 | 1 | 0 |
| 359 | t359 | CC359/938 |  | 07-23-12-21-17-34-34-33-34 | 1 | 0 | 1 | 0 |
| 359 | t521 | CC359/938 |  | 07-23-12-21-17-34-34-34-34-33-34 | 1 | 0 | 1 | 0 |
| 359 | t938 | CC359/938 |  | 07-23-12-21-17-34-34-33-34-34 | 1 | 3 | 0 | 0 |
| 359 | t3992 | CC359/938 |  | 26-23-12-21-17-34-34-33-34 | 1 | 0 | 1 | 0 |
| 370 | t370 | CC21 | CC30 | 09-34-17-34-16-34 | 2 | 3 | 0 | 0 |
| 370 | t2143 | CC21 | CC30 | 09-13-17-34-16-34 | 1 | 0 | 4 | 0 |
| 375 | t375 | CC375 |  | 49-13-23-05-17-34-33-34 | 1 | 8 | 0 | 0 |
| 375 | t525 | CC525 |  | 49-34-23-05-17-34-33-34 | 1 | 4 | 0 | 0 |
| 442 | t010 | CC2 |  | 26-17-34-17-20-17-12-17-16 | 3 | 4 | 6 | 0 |
| 442 | t442 | CC2 |  | 35-17-34-17-20-17-12-17-16 | 1 | 6 | 0 | 0 |
| 571 | t571 | CC21 | CC30 | 08-16-02-25-02-25-34-25 | 7 | 15 | 16 | 0 |
| 571 | t1451 | CC21 | CC30 | 08-16-02-25-34-25 | 2 | 4 | 3 | 0 |
| 571 | t3085 | CC21 | CC30 | 08-16-02-25-02-25-34-25-34-25 | 1 | 0 | 4 | 0 |
| 571 | t5635 | CC21 | CC30 | 08-16-02-25-02-25-34-34-25 | 1 | 0 | 1 | 0 |
| 579 | t579 | CC2 |  | 26-23-17-17-20-17-12-17-16 | 1 | 12 | 0 | 0 |
| 608 | t310 | CC5 | CC22 | 26-23-31-05-17-25-17-25-16-28 | 1 | 2 | 0 | 0 |
| 608 | t608 | CC5 | CC22 | 26-23-31-29-17-25-17-25-16-28 | 1 | 1 | 0 | 1 |
| 620 | t620 | CC21 | CC30 | 08-16-02-16-34-13-17-34 | 1 | 17 | 0 | 0 |
| 620 | t1078 | CC21 | CC30 | 08-16-02-16-34-13-17-34-34 | 1 | 1 | 0 | 0 |
| 710 | t710 | CC21 | CC30 | 15-12-16-02-16-02-24 | 1 | 16 | 0 | 0 |
| 803 | t803 | CC84 | CC15 | 07-23-02-12-23 | 1 | 6 | 0 | 0 |
| 954 | t954 | CC2 |  | 26-23-17-34-17-17-16 | 1 | 14 | 0 | 0 |
| 975 | t975 | CC21 | CC30 | 08-16-02-16-02-25-17 | 2 | 15 | 0 | 0 |
| 1046 | t195 | CC164 |  | 07-06-17-21-34-34 | 1 | 1 | 0 | 0 |
| 1046 | t1046 | CC164 |  | 07-06-17-21-34-34-34 | 1 | 1 | 0 | 0 |
| 1120 | t6899 | CC5 | CC22 | 35-13-23-31-05-05-17-25-17-25-16-28 | 1 | 1 | 0 | 0 |
| 1149 | t1149 | CC1149/1614 |  | 08-16-34-24-34-17-17 | 1 | 3 | 0 | 0 |
| 1541 | t280 | CC280 |  | 04-20-17-12-12-17 | 1 | 12 | 0 | 0 |
| 1541 | t1541 | CC280 |  | 04-20-17-12-17-17 | 2 | 0 | 11 | 0 |
| 1996 | t031 | CC21 | CC30 | 08-16-02-16-34-13-34-16-34 | 2 | 10 | 0 | 0 |
| 2133 | t888 | CC160 |  | 07-23-21-24-33-17 | 3 | 20 | 0 | 0 |
| 2133 | t2133 | CC160 |  | 07-23-21-33-22-17 | 1 | 0 | 3 | 0 |
| 3092 | t148 | CC148 |  | 07-23-12-21-12-17-20-17-12-12-17 | 2 | 6 | 0 | 0 |
| 3092 | t3092 | CC3092 |  | 07-23-12-21-12-17-20-17-12-17 | 1 | 14 | 0 | 0 |
| 3240 | t3240 | CC24 | CC8 | 11-21-17-34-22-25 | 2 | 2 | 0 | 0 |
| 3240 | t6940 | CC24 | CC8 | 11-10-17-34-22-25 | 1 | 0 | 1 | 0 |
| 3879 | t2643 | CC21 | CC30 | 08-16-16-02-17-24-24 | 2 | 11 | 0 | 0 |
| 3879 | t3879 | CC21 | CC30 | 08-16-16-02-17-24 | 1 | 2 | 0 | 0 |
| 5133 | t334 | CC24 | CC8 | 11-12-21-17-34-22-25 | 1 | 0 | 1 | 0 |
| 5133 | t5133 | CC24 | CC8 | 11-10-21-17-34-34-22-25 | 1 | 0 | 6 | 0 |
| 6416 | t6416 | CC078/349 |  | 04-12-21-12-17-20-17-12-12-12-17 | 1 | 9 | 0 | 0 |
| 6416 | t6856 | CC078/349 |  | 04-12-21-12-17-20-17-12-12-12-12-17 | 1 | 9 | 0 | 0 |
| 6807 | t871 | CC21 | CC30 | 15-12-16-17-24-24 | 1 | 1 | 0 | 0 |
| 6807 | t6807 | CC21 | CC30 | 15-12-25-17-24-24 | 1 | 7 | 0 | 0 |
| 6855 | t6855 | CC21 | CC30 | 15-12-16-02-16-02-31-31-25-17-24-24 | 1 | 8 | 0 | 0 |
| 7855 | t7855 | CC21 | CC30 | 15-12-02-16-02-24-24-24 | 1 | 0 | 3 | 0 |
| 9383 | t9383 | Singleton |  | 08-16-34-02-43-34-16-16-16-16-16-16-16-16-16-17-16 | 1 | 2 | 0 | 0 |
| 004/330 | t004 | CC21 | CC30 | 09-02-16-13-13-17-34-16-34 | 1 | 1 | 0 | 0 |
| 004/330 | t040 | CC21 | CC30 | 09-02-16-13-17-34-16-34 | 1 | 8 | 0 | 0 |
| 004/330 | t065 | CC21 | CC30 | 09-02-16-34-13-17-34-16-34 | 5 | 32 | 0 | 0 |
| 004/330 | t330 | CC21 | CC30 | 09-02-16-34-34-17-34-16-34 | 1 | 0 | 3 | 0 |
| 004/330 | t350 | CC21 | CC30 | 09-02-16-34-42-17-34-16-34 | 1 | 14 | 0 | 0 |
| 004/330 | t5211 | CC21 | CC30 | 09-02-16-17-34-16-34 | 1 | 4 | 0 | 0 |
| 004/330 | t7871 | CC21 | CC30 | 09-02-16-34-02-16-34-34-17-34-16-34 | 1 | 1 | 0 | 0 |
| 012/021 | t012 | CC21 | CC30 | 15-12-16-02-16-02-25-17-24-24 | 41 | 288 | 27 | 7 |
| 012/021 | t017 | CC21 | CC30 | 15-12-16-16-02-16-02-25-17-24-24 | 1 | 18 | 0 | 0 |
| 012/021 | t018 | CC21 | CC30 | 15-12-16-02-16-02-25-17-24-24-24 | 4 | 32 | 0 | 11 |
| 012/021 | t021 | CC21 | CC30 | 15-12-16-02-16-02-25-17-24 | 19 | 89 | 3 | 2 |
| 012/021 | t037 | CC21 | CC30 | 15-12-16-02-25-17-24 | 3 | 38 | 0 | 0 |
| 012/021 | t096 | CC21 | CC30 | 15-12-16-16-02-17-24 | 2 | 11 | 0 | 0 |
| 012/021 | t219 | CC21 | CC30 | 15-12-16-16-02-17-24-24-24 | 1 | 14 | 0 | 0 |
| 012/021 | t275 | CC21 | CC30 | 15-12-16-02-25-17-24-24 | 1 | 7 | 0 | 0 |
| 012/021 | t298 | CC21 | CC30 | 15-12-16-02-17-24 | 1 | 2 | 0 | 0 |
| 012/021 | t318 | CC21 | CC30 | 15-12-16-16-02-16-02-25-17-24 | 2 | 13 | 0 | 0 |
| 012/021 | t338 | CC21 | CC30 | 15-21-16-02-25-17-24 | 6 | 34 | 0 | 0 |
| 012/021 | t342 | CC21 | CC30 | 15-12-16-02-16-02-25-17 | 2 | 25 | 0 | 0 |
| 012/021 | t382 | CC21 | CC30 | 15-12-16-16-02-17-24-24 | 4 | 26 | 4 | 0 |
| 012/021 | t399 | CC21 | CC30 | 15-12-16-02-17-24-24 | 2 | 13 | 0 | 0 |
| 012/021 | t789 | CC21 | CC30 | 15-12-16-02-16-02-17-24-24 | 1 | 10 | 0 | 0 |
| 012/021 | t1239 | CC21 | CC30 | 15-12-16-02-16-02-24-24-24 | 3 | 11 | 0 | 0 |
| 012/021 | t1306 | CC21 | CC30 | 15-12-16-02-16-02-16-02-25-17-24-24 | 1 | 9 | 0 | 0 |
| 012/021 | t1414 | CC21 | CC30 | 15-12-16-02-16-02-25-24 | 2 | 1 | 1 | 0 |
| 012/021 | t1626 | CC21 | CC30 | 15-12-16-02-16-34-17-24-24 | 1 | 0 | 11 | 0 |
| 012/021 | t2271 | CC21 | CC30 | 15-12-16-16-02-25-17-24-24 | 2 | 22 | 0 | 0 |
| 012/021 | t2868 | CC21 | CC30 | 15-21-21-16-02-25-17-24 | 1 | 6 | 0 | 0 |
| 012/021 | t3284 | CC21 | CC30 | 15-12-16-16-16-02-17-24-24 | 1 | 3 | 0 | 0 |
| 012/021 | t3732 | CC21 | CC30 | 15-21-16-02-25-17-24-24 | 1 | 14 | 0 | 0 |
| 012/021 | t4451 | CC21 | CC30 | 15-12-23-16-02-25-17-24-24-24 | 1 | 6 | 0 | 0 |
| 012/021 | t6584 | CC21 | CC30 | 15-12-16-34-16-02-25-17-24 | 1 | 12 | 0 | 0 |
| 012/021 | t6803 | CC21 | CC30 | 15-12-16-02-16-02-25-17-24-24-17-24 | 2 | 20 | 0 | 0 |
| 012/021 | t6815 | CC21 | CC30 | 15-21-12-16-02-25-17-24-24 | 1 | 4 | 0 | 0 |
| 122/019 | t019 | CC21 | CC30 | 08-16-02-16-02-25-17-24 | 2 | 5 | 0 | 0 |
| 122/019 | t122 | CC21 | CC30 | 08-16-02-16-02-25-17-24-24 | 2 | 0 | 4 | 0 |
| 122/019 | t363 | CC21 | CC30 | 15-16-02-25-17-24 | 1 | 4 | 0 | 0 |
| 122/019 | t6134 | CC21 | CC30 | 15-16-02-16-02-25-17-24-24-24 | 1 | 12 | 0 | 0 |
| 186/786 | t186 | CC186 |  | 07-12-21-17-13-13-34-34-33-34 | 4 | 10 | 0 | 0 |
| 186/786 | t786 | CC186 |  | 07-12-21-17-13-34-34-33-34 | 1 | 1 | 0 | 0 |
| 186/786 | t5973 | CC186 |  | 07-12-21-17-13-13-13-13-13-34-34-33-13 | 1 | 0 | 1 | 0 |
| 186/786 | t8868 | CC186 |  | 07-12-21-17-13-34-34-481-34 | 1 | 0 | 1 | 0 |
| 659/408 | t171 | CC659 |  | 14-44-13-12-17-17-17-17-23-18 | 6 | 38 | 0 | 0 |
| 659/408 | t408 | CC659 |  | 14-44-13-12-17-17-17-17-17-23-18 | 3 | 26 | 0 | 0 |
| 659/408 | t659 | CC659 |  | 14-44-13-12-17-17-17-23-18 | 3 | 10 | 1 | 0 |
| 659/408 | t6417 | CC659 |  | 14-44-13-12-17-13-12-17-17-17-23-18 | 1 | 12 | 0 | 0 |
| 659/408 | t6795 | CC659 |  | 14-44-13-12-17-17-17-17-17-22-18 | 1 | 12 | 0 | 0 |
| Excluded1 | t3345 | Excluded |  | 26-23-12-23 | 1 | 14 | 0 | 0 |
| Excluded10 | t3369 | Excluded |  | 14-44 | 1 | 5 | 0 | 0 |
| Excluded11 | t870 | Excluded |  | 15/12/2017 | 1 | 12 | 0 | 0 |
| Excluded12 | t528 | Excluded |  | 4 | 3 | 6 | 2 | 0 |
| Excluded14 | t4776 | Excluded |  | 04/12/2016 | 1 | 6 | 0 | 0 |
| Excluded15 | t748 | Excluded |  | 15-12-17-24 | 2 | 1 | 1 | 0 |
| Excluded16 | t643 | Excluded |  | 04-21-12-17 | 1 | 1 | 0 | 0 |
| Excluded17 | t026 | Excluded |  | 08-16-34 | 5 | 8 | 4 | 0 |
| Excluded19 | t398 | Excluded |  | 26-33-13 | 1 | 3 | 0 | 0 |
| Excluded2 | t779 | Excluded |  | 8 | 2 | 4 | 0 | 0 |
| Excluded21 | t8811 | Excluded |  | 04-254-22-25 | 1 | 2 | 0 | 0 |
| Excluded22 | t693 | Excluded |  | 7 | 1 | 1 | 0 | 0 |
| Excluded23 | t386 | Excluded |  | 07-23-13 | 1 | 2 | 0 | 0 |
| Excluded24 | t2726 | Excluded |  | 164-34-16-34 | 2 | 3 | 0 | 0 |
| Excluded25 | t1192 | CC12 |  | 15 | 1 | 1 | 0 | 0 |
| Excluded3 | t1509 | Excluded |  | 07-23-12-23 | 2 | 10 | 0 | 0 |
| Excluded4 | t1544 | Excluded |  | 07-22-34 | 2 | 12 | 0 | 0 |
| Excluded5 | t1040 | Excluded |  | 08-16-34-34 | 1 | 13 | 0 | 0 |
| Excluded6 | t1445 | Excluded |  | 121-17-02-24 | 1 | 15 | 0 | 0 |
| Excluded7 | t605 | Excluded |  | Jul-23 | 2 | 33 | 0 | 0 |
| Excluded8 | t2365 | Excluded |  | 04-20-16-34 | 1 | 19 | 0 | 0 |
| Excluded9 | t227 | Excluded |  | 04-12-12-17 | 2 | 15 | 0 | 0 |
| s1 | t045 | CC2 |  | 26-17-20-17-12-17-16 | 3 | 28 | 0 | 0 |
| s101 | t8585 | Singleton |  | 26-23-20-17-31-17-31 | 1 | 2 | 0 | 0 |
| s102 | t9584 | Singleton |  | 07-23-12-12-12-12 | 1 | 1 | 0 | 0 |
| s105 | t6801 | CC32 |  | 26-23-13-23-31-29-17-25-25-16-28 | 1 | 1 | 0 | 0 |
| s107 | t773 | Singleton |  | 04-44-33-31-16-12-25-22-34 | 1 | 1 | 0 | 0 |
| s109 | t1236 | CC186 |  | 26-23-12-21-17-34-34-34-33-34 | 1 | 1 | 0 | 0 |
| s11 | t364 | CC364 |  | 04-34-17-32-17-23-24 | 7 | 36 | 0 | 0 |
| s110 | t3319 | CC32 |  | 26-23-16-23-31-29-17-25-17-25-16-28 | 1 | 1 | 0 | 0 |
| s112 | t6825 | Singleton |  | 26-23-13-23-31-31 | 1 | 1 | 0 | 0 |
| s113 | t6826 | CC12 |  | 15-12-16-34-02-16-02-24-24-24 | 1 | 1 | 0 | 0 |
| s12 | t377 | CC24 | CC8 | 04-02-12-21-17-34-22-25 | 2 | 1 | 3 | 0 |
| s13 | t400 | CC24 | CC8 | 11-19-12-21-17-34-34-22-25 | 2 | 0 | 2 | 0 |
| s14 | t493 | CC364 |  | 04-34-17-66-32-17-23-24 | 3 | 6 | 2 | 0 |
| s15 | t505 | CC30 |  | 08-02-16-34-13-17-34-16-34 | 2 | 20 | 0 | 0 |
| s16 | t530 | CC24 | CC8 | 11-19-12-21-17-34-24-34-16 | 1 | 9 | 0 | 0 |
| s18 | t537 | Singleton |  | 07-23-12-21-12-20-17-12-12 | 2 | 1 | 3 | 0 |
| s19 | t575 | CC2 |  | 26-23-17-12-17-17-16 | 1 | 17 | 0 | 0 |
| s2 | t273 | Singleton |  | 07-23-21-17-13-34-16-34-33-13 | 1 | 10 | 0 | 0 |
| s21 | t737 | CC21 | CC30 | 08-16-34-34-13-17-34-16-34 | 1 | 13 | 0 | 0 |
| s23 | t817 | CC136 |  | 04-54-31-12-16-34-16-12-25-22-22-34 | 1 | 3 | 0 | 0 |
| s24 | t853 | CC84 | CC15 | 07-23-34-34-12-12-23-02-12-23 | 1 | 0 | 1 | 0 |
| s25 | t922 | CC127 |  | 07-23-21-16-33-13 | 2 | 0 | 6 | 0 |
| s26 | t939 | Singleton |  | 04-16-34-12-34-12 | 1 | 1 | 0 | 0 |
| s28 | t1070 | CC21 | CC30 | 15-12-16-34-02-25-17-24-24 | 1 | 12 | 0 | 0 |
| s3 | t9726 | Unknown |  | 07-23-21-17-34-34-12-23 | 1 | 0 | 2 | 0 |
| s30 | t1166 | Singleton |  | 03-16-21-17-23-13-17-17-17-23-24 | 1 | 0 | 5 | 0 |
| s32 | t1265 | CC2 |  | 26-23-17-34-17-20-17-12-12-12-16 | 1 | 11 | 0 | 0 |
| s33 | t1347 | CC21 | CC30 | 08-02-16-02-25-17-24 | 1 | 0 | 3 | 0 |
| s34 | t1596 | CC659 |  | 14-44-13-12-17-18-17 | 1 | 1 | 0 | 0 |
| s37 | t1720 | CC21 | CC30 | 08-16-16-02-25-17-24-24 | 1 | 14 | 0 | 0 |
| s38 | t140 | CC280 |  | 04-21-12-12-17 | 1 | 13 | 0 | 0 |
| s39 | t1736 | Singleton |  | 04-82-17-25-16-17 | 1 | 0 | 1 | 0 |
| s40 | t1827 | CC21 | CC30 | 15-12-17-24-24 | 1 | 1 | 0 | 0 |
| s41 | t1987 | CC164 |  | 07-06-17-21-34 | 2 | 15 | 0 | 0 |
| s42 | t2029 | CC21 | CC30 | 15-12-02-25-17-24 | 1 | 1 | 0 | 0 |
| s43 | t2292 | CC078/349 |  | 04-20-12-17-20-17 | 1 | 4 | 0 | 0 |
| s45 | t2883 | CC127 |  | 07-23-21-17-34 | 1 | 14 | 0 | 0 |
| s47 | t3262 | CC84 | CC15 | 14-12-34-34-12-12-23 | 2 | 8 | 0 | 0 |
| s49 | t190 | CC24 | CC8 | 11-17-34-24-34-22-25 | 3 | 10 | 1 | 0 |
| s5 | t9794 | Unknown |  | 11-12-16-34-16-12-25 | 1 | 1 | 0 | 0 |
| s50 | t3610 | Singleton |  | 35-17-25-16-28 | 1 | 10 | 0 | 0 |
| s54 | t4322 | CC136 |  | 04-44-33-31-12-16-34-12-25-22-22-34 | 1 | 6 | 0 | 0 |
| s55 | t4549 | CC4549 |  | 04-34-21-17-21-17-34-22-25 | 1 | 2 | 0 | 0 |
| s56 | t4553 | CC4553 |  | 04-34-21-17-34-21-22-25 | 1 | 2 | 0 | 0 |
| s57 | t4714 | CC84 | CC15 | 07-23-12-34-23-02-12-23 | 1 | 7 | 0 | 0 |
| s58 | t4751 | CC21 | CC30 | 08-23-16-34-13-17-34 | 1 | 1 | 0 | 0 |
| s60 | t196 | CC364 |  | 04-34-32-17-23-24 | 2 | 8 | 0 | 0 |
| s63 | t5855 | CC84 | CC15 | 07-23-44-34-34-12-12-23-02-12-23 | 1 | 7 | 0 | 0 |
| s64 | t6154 | Singleton |  | 04-20-17-17-31-31-24-17-17-17-17-17-17-25 | 1 | 4 | 0 | 0 |
| s65 | t6292 | Singleton |  | 14-44-12-17-23-18-110-17-17-17-23-24 | 1 | 0 | 1 | 0 |
| s66 | t6390 | CC89 |  | 04-33-31-12-16-34-16-12 | 2 | 21 | 0 | 0 |
| s67 | t6791 | CC89 |  | 04-33-31-12-16-34-16-21-34 | 2 | 15 | 0 | 0 |
| s68 | t6792 | Singleton |  | 08-16-02-16-17-13-17-13-17-16-34 | 1 | 16 | 0 | 0 |
| s69 | t6794 | Singleton |  | 11-12-16-34-16-12-25-22-22-34 | 1 | 9 | 0 | 0 |
| s70 | t6796 | CC24 | CC8 | 11-21-17-36-34-22-25 | 2 | 17 | 0 | 0 |
| s71 | t209 | CC127 |  | 07-16-12-23-34 | 6 | 50 | 0 | 0 |
| s72 | t6797 | CC24 | CC8 | 11-10-34-24-34-22-25-25 | 1 | 18 | 0 | 0 |
| s73 | t6800 | Singleton |  | 07-34-16-34-31-13 | 1 | 4 | 0 | 0 |
| s74 | t6804 | CC84 | CC15 | 07-16-34-34-12-12-23 | 1 | 14 | 0 | 0 |
| s75 | t6805 | Singleton |  | 04-20-17-23-20-17-24 | 1 | 2 | 0 | 0 |
| s76 | t6806 | CC6806 |  | 04-44-33-25-22-22-34 | 1 | 7 | 0 | 0 |
| s77 | t6808 | CC21 | CC30 | 08-16-02-16-16-16-34 | 1 | 5 | 0 | 0 |
| s78 | t6809 | CC24 | CC8 | 11-10-21-17-34-24-204-22-25 | 1 | 8 | 0 | 0 |
| s79 | t6810 | CC24 | CC8 | 398-10-21-17-34-24-34-22-25 | 1 | 3 | 0 | 0 |
| s80 | t6811 | CC127 |  | 07-23-21-16-34-33 | 1 | 16 | 0 | 0 |
| s81 | t6814 | Singleton |  | 07-83-06-17-21-34-34-22-194 | 1 | 8 | 0 | 0 |
| s82 | t213 | CC160 |  | 07-23-12-21-24-33-22-17 | 3 | 37 | 0 | 0 |
| s83 | t6816 | CC280 |  | 04-12-13-20-17-12-12-17 | 1 | 1 | 0 | 0 |
| s84 | t6820 | Singleton |  | 08-16-16-51-68-02-24-02-24 | 2 | 1 | 2 | 0 |
| s85 | t6821 | Singleton |  | 07-23-02-34-23-02-34-34 | 1 | 15 | 0 | 0 |
| s86 | t6823 | Singleton |  | 04-44-24-12-16-34-188-12-25-22-34 | 1 | 4 | 0 | 0 |
| s87 | t6828 | Singleton |  | 04-02-12-12-21-17-34-21-17-21-17-34-22-25 | 1 | 3 | 0 | 0 |
| s88 | t6829 | CC1149/1614 |  | 08-16-34-24-17-17-17 | 2 | 6 | 0 | 0 |
| s89 | t6833 | Singleton |  | 04-82-17-25-17-25-24 | 1 | 1 | 0 | 0 |
| s9 | t337 | Singleton |  | 07-16-23-23-02-12-23-02-34 | 2 | 19 | 0 | 0 |
| s90 | t6835 | CC84 | CC15 | 07-23-21-22-17-34-12-23-02-12-23 | 1 | 0 | 2 | 0 |
| s91 | t6854 | CC1149/1614 |  | 07-34-24-17-17-17 | 1 | 2 | 0 | 0 |
| s92 | t6941 | CC84 | CC15 | 07-23-12-34-34-12-12-23-02-23-02-02-12-23 | 1 | 10 | 0 | 0 |
| s94 | t7050 | CC216 |  | 04-02-17-12-31-16-34 | 1 | 5 | 0 | 0 |
| s95 | t7060 | CC21 | CC30 | 15-12-16-16-02-17-25-16-02-17-24-24 | 1 | 0 | 9 | 0 |
| s96 | t7205 | CC359/938 |  | 07-23-13-21-17-34-34-34-33-02-34 | 1 | 1 | 0 | 0 |
| s97 | t7960 | Singleton |  | 299-25-17-17-16-16-16-16 | 1 | 1 | 0 | 0 |

*spa*-types which could not be assigned a CC are marked as excluded

**Supplementary Table 5: Univariable impact of participant risk factors on time to acquisition of a new *spa*-type or loss of a *S. aureus* *spa*-type.**

**(a) Participant characteristics and behaviour at recruitment**

|  | *(Effect in Cox regression)* | Number acquire (N=162) n (row %) or median (IQR) | Number do not acquire (N=382) n (row %) or median (IQR) | Univariable time to acquisition Hazard Ratio | Time to acquisition *P* value* | Number lose (N=231) n (%) or median (IQR) | Number do not lose (N=198) n (%) or median (IQR) | Univariable time to loss Hazard Ratio | Time to loss *P* value* |
| --- | --- | --- | --- | --- | --- | --- | --- | --- | --- |
| *S. aureus* details | | | | | | | | | |
| Recruitment- | *Yes* | 63 (32%) | 135 (68%) | 1.27 | 0.14 | 55 (81%) | 13 (19%) | 3.17 | <0.0001 |
| negative | *No* | 99 (29%) | 247 (71%) | 1.00 |  | 176 (49%) | 185 (51%) | 1.00 |  |
| *spa*-CC present at recruitment | CC30 (*spa-CC021)* | 23 (22%) | 80 (78%) | 1.00 | 0.007 | 48 (47%) | 55 (53%) | 1.00 | 0.39 |
|  | CC15 (*spa-CC084)* | 16 (38%) | 26 (62%) | 2.08 |  | 22 (52%) | 20 (48%) | 1.22 |  |
|  | *spa-CC002* | 6 (27%) | 16 (73%) | 1.20 |  | 12 (55%) | 10 (45%) | 1.31 |  |
|  | CC22 *(spa-CC005)* | 7 (29%) | 17 (71%) | 1.27 |  | 7 (29%) | 17 (71%) | 0.54 |  |
|  | *spa-CC127* | 7 (33%) | 14 (67%) | 1.30 |  | 14 (67%) | 7 (33%) | 1.31 |  |
|  | *spa-CC160* | 3 (21%) | 11 (79%) | 1.00 |  | 7 (50%) | 7 (50%) | 1.12 |  |
|  | CC8 *(spa-CC024)* | 8 (62%) | 5 (38%) | 5.19 |  | 9 (69%) | 4 (31%) | 1.63 |  |
|  | *Other CC* | 29 (27%) | 78 (73%) | 1.29 |  | 57 (53%) | 50 (47%) | 1.30 |  |
| Participant characteristics | | | | | | | | | |
| Age | *Per 10 years older* | 40 (20;61) | 53 (30;65) | 0.79 | <0.0001 | 44 (21;62) | 57 (37;68) | 0.83 | <0.0001 |
| Sex | *Female* | 84 (29%) | 203 (71%) | 1.00 |  | 128 (57%) | 98 (43%) | 1.00 |  |
|  | *Male* | 78 (30%) | 179 (70%) | 1.07 | 0.67 | 103 (51%) | 100 (49%) | 0.81 | 0.10 |
| Student | *No* | 116 (27%) | 318 (73%) | 1.00 |  | 178 (50%) | 175 (50%) | 1.00 |  |
|  | *Yes* | 46 (46%) | 64 (64%) | 2.35 | <0.0001 | 53 (70%) | 23 (30%) | 1.33 | 0.11 |
| Ethnic background | *White British/Irish* | 150 (30%) | 343 (70%) | 1.00 | 1.00 | 211 (56%) | 168 (44%) | 1.00 | 0.11 |
|  | *Other white* | 8 (25%) | 24 (75%) | 0.99 |  | 10 (45%) | 12 (55%) | 0.82 |  |
|  | *Other ethnicity* | 4 (21%) | 15 (79%) | 1.09 |  | 10 (77%) | 3 (23%) | 1.90 |  |
| Participant behaviour | | | | | | | | | |
| Current employment | *No* | 82 (28%) | 208 (72%) | 1.00 |  | 117 (52%) | 108 (48%) | 1.00 |  |
|  | *Yes* | 80 (31%) | 174 (69%) | 1.04 | 0.83 | 114 (56%) | 90 (44%) | 1.02 | 0.91 |
| Current healthcare | *No* | 127 (30%) | 295 (70%) | 1.00 |  | 170 (50%) | 169 (50%) | 1.00 |  |
| related employment | *Yes* | 35 (29%) | 87 (71%) | 0.88 | 0.50 | 61 (68%) | 29 (32%) | 1.43 | 0.02 |
| Number of other household members | *Lives alone* | 15 (21%) | 55 (79%) | 1.00 | 0.0001 | 27 (47%) | 31 (53%) | 1.00 | <0.0001 |
|  | *1 household member* | 55 (25%) | 167 (75%) | 1.17 |  | 75 (47%) | 85 (53%) | 1.05 |  |
|  | *2/3 household members* | 68 (35%) | 124 (65%) | 2.12 |  | 94 (62%) | 57 (38%) | 1.82 |  |
|  | *4 or more* | 19 (37%) | 32 (63%) | 2.62 |  | 29 (78%) | 8 (22%) | 2.60 |  |
|  | *Shared accommodation* | 5 (56%) | 4 (44%) | 5.16 |  | 6 (75%) | 2 (25%) | 2.74 |  |
| Number of other household members with healthcare contact | *Zero* | 135 (30%) | 313 (70%) | 1.00 | 0.19 | 192 (56%) | 149 (44%) | 1.00 | 0.26 |
|  | *One* | 22 (25%) | 65 (75%) | 0.81 |  | 33 (51%) | 32 (49%) | 0.92 |  |
|  | *Two or more* | 5 (56%) | 4 (44%) | 1.98 |  | 6 (75%) | 2 (25%) | 1.90 |  |
| Current participation | *No* | 94 (27%) | 252 (73%) | 1.00 |  | 136 (50%) | 136 (50%) | 1.00 |  |
| in contact sport | *Yes* | 68 (34%) | 130 (66%) | 1.24 | 0.17 | 95 (61%) | 62 (39%) | 1.27 | 0.08 |
| Looks after anyone | *No* | 126 (39%) | 195 (61%) | 1.00 |  | 178 (54%) | 152 (46%) | 1.00 |  |
| with a disability/old age | *Yes* | 36 (29%) | 87 (71%) | 0.89 | 0.53 | 53 (54%) | 46 (46%) | 0.89 | 0.44 |

**(b) Participant previous healthcare exposure and co-morbidities at recruitment**

|  | *(Effect in Cox regression)* | Number acquire (N=157) n (row %) or median (IQR) | Number do not acquire (N=364) n (row %) or median (IQR) | Univariable time to acquisition Hazard Ratio | Time to acquisition *P* value* | Number lose (N=220) n (%) or median (IQR)† | Number do not lose (N=181) n (%) or median (IQR) | Univariable time to loss Hazard Ratio | Time to loss *P* value* |
| --- | --- | --- | --- | --- | --- | --- | --- | --- | --- |
| Previous healthcare exposure | | | | | | | | | |
| Ever been an inpatient | *No* | 42 (41%) | 60 (59%) | 1.00 |  | 47 (59%) | 32 (41%) | 1.00 |  |
|  | *Yes* | 115 (27%) | 304 (73%) | 0.58 | 0.002 | 173 (54%) | 149 (46%) | 0.80 | 0.18 |
| Days since in last inpatient episode | *Per year* | 986 (345;3289) | 1151 (382;3486) | 0.99 | 0.60 | 1130 (382;3289) | 1000 (339;2897) | 1.02 | 0.38 |
| Ever been an outpatient | *No* | 7 (28%) | 18 (72%) | 1.00 |  | 8 (36%) | 14 (64%) | 1.00 |  |
|  | *Yes*  *Unknown†* | 150 (30%)  *0* | 346 (70%)  *1* | 1.10 | 0.81 | 212 (56%) | 167 (44%) | 1.71 | 0.14 |
| Days since last outpatient appointment ** | *Per year* | 322 (97;921) | 299 (86;1106) | 1.00 | 0.98 | 324 (106;900) | 294 (84;1174) | 0.97 | 0.17 |
| Ever had a GP | *No* | 1 (33%) | 2 (67%) | 1.00 |  | 2 (67%) | 1 (33%) | 1.00 |  |
| appointment before recruitment | *Yes* | 156 (30%) | 362 (70%) | 1.04 | 0.97 | 218 (55%) | 180 (45%) | 1.20 | 0.80 |
| Days since last GP appointment ** | *Per year* | 77 (28;213) | 68 (25;179) | 1.27 | 0.02 | 68 (28;181) | 63 (23;170) | 1.01 | 0.87 |
| Ever had a practice | *No* | 5 (42%) | 7 (58%) | 1.00 |  | 8 (73%) | 3 (27%) | 1.00 |  |
| nurse appointment | *Yes*  *Unknown†* | 152 (30%)  *0* | 357 (70%)  *2* | 0.59 | 0.25 | 212 (54%)  *1* | 178 (46%)  *0* | 0.61 | 0.18 |
| Days since last practice nurse appointment ** | *Yes (vs no)* | 144 (48;381) | 98 (27;309) | 1.07 | 0.28 | 116 (34;371) | 119 (29;308) | 1.07 | 0.14 |
| Ever had a district nurse | *No* | 136 (31%) | 306 (69%) | 1.00 |  | 183 (54%) | 154 (46%) | 1.00 |  |
| appointment | *Yes*  *Unknown†* | 21 (27%)  *3* | 58 (73%)  *4* | 0.81 | 0.38 | 37 (58%)  *3* | 27 (42%)  *3* | 0.99 | 0.95 |
| Days since last district nurse appointment | *Per year* | 1812 (462;4636) | 2843 (1106;5609) | 0.92 | 0.13 | 2388 (679;6693) | 3287 (1106;5609) | 0.96 | 0.39 |
| Co-morbidities | | | | | | | | | |
| Has a long-term illness | *No* | 83 (33%) | 169 (67%) | 1.00 |  | 110 (61%) | 71 (39%) | 1.00 |  |
|  | *Yes* | 74 (28%) | 195 (72%) | 0.70 | 0.03 | 110 (50%) | 110 (50%) | 0.67 | 0.004 |
| Ever had chemotherapy | *No* | 154 (30%) | 354 (70%) | 1.00 |  | 214 (54%) | 179 (46%) | 1.00 |  |
|  | *Yes*  *Unknown†* | 3 (23%)  *0* | 10 (77%)  *2* | 0.68 | 0.51 | 6 (75%)  *1* | 2 (25%)  *0* | 1.30 | 0.53 |
| Ever had renal dialysis | *No* | 156 (30%) | 362 (70%) | 1.00 |  | 218 (55%) | 180 (45%) | 1.00 |  |
|  | *Yes*  *Unknown†* | 1 (33%)  *0* | 2 (67%)  *1* | 1.42 | 0.73 | 2 (67%)  *0* | 1 (33%)  *3* | 1.40 | 0.64 |
| Ever had surgery | *No* | 56 (39%) | 88 (61%) | 1.00 |  | 60 (55%) | 49 (45%) | 1.00 |  |
|  | *Yes*  *Unknown†* | 101 (27%)  *0* | 276 (73%)  *1* | 0.57 | 0.001 | 160 (55%)  *1* | 132 (45%)  *0* | 0.91 | 0.52 |
| Days since surgery | *Per year* | 2894 (671;5808) | 2571 (824;6077) | 1.00 | 0.89 | 2510 (884;5677) | 3281 (921;6829) | 0.98 | 0.38 |
| Ever prescribed oral | *No* | 132 (30%) | 311 (70%) | 1.00 |  | 187 (55%) | 155 (45%) | 1.00 |  |
| steroids | *Yes*  *Unknown†* | 25 (32%)  *1* | 53 (68%)  *10* | 1.09 | 0.71 | 33 (56%)  *2* | 26 (44%)  *3* | 0.99 | 0.98 |
| Treatment for skin | *Yes (vs no)* | 139 (30%) | 317 (70%) | 1.00 |  | 191 (55%) | 159 (45%) | 1.00 |  |
| conditions in last 30 days | *Yes (vs no)*  *Unknown†* | 18 (28%)  *2* | 47 (72%)  *3* | 0.93 | 0.78 | 29 (57%)  *0* | 22 (43%)  *5* | 1.01 | 0.94 |
| Ever had vascular | *No* | 64 (34%) | 122 (66%) | 1.00 |  | 81 (57%) | 61 (43%) | 1.00 |  |
| access | *Yes*  *Unknown†* | 93 (28%)  *6* | 242 (72%)  *10* | 0.75 | 0.08 | 139 (54%)  *8* | 120 (46%)  *8* | 0.93 | 0.58 |
| Ever had a catheter | *No* | 119 (30%) | 281 (70%) | 1.00 |  | 164 (52%) | 150 (48%) | 1.00 |  |
|  | *Yes*  *Unknown†* | 38 (31%)  *5* | 83 (69%)  *13* | 0.99 | 0.94 | 56 (64%)  *7* | 31 (36%)  *11* | 1.16 | 0.34 |
| Ever prescribed | *No* | 8 (35%) | 15 (65%) | 1.00 |  | 9 (50%) | 9 (50%) | 1.00 |  |
| antibiotics  *Unknown* | *Yes (vs no)* | 149 (30%)  *3* | 349 (70%)  *7* | 0.76 | 0.45 | 211 (55%)  *4* | 172 (45%)  *2* | 1.10 | 0.77 |
| Days since last antibiotic prescribed ** | *Per year* | 467 (169;1833) | 631 (184;2043) | 1.00 | 0.84 | 489 (160;1859) | 718 (206;2089) | 0.99 | 0.47 |
| Had MRSA previously | *No* | 155 (30%) | 361 (70%) | 1.00 |  | 219 (55%) | 178 (45%) | 1.00 |  |
|  | *Yes*  *Unknown†* | 2 (40%)  *2* | 3 (60%)  *13* | 1.70 | 0.46 | 1 (25%)  *3* | 3 (75%)  *4* | 0.42 | 0.38 |
| Had MSSA previously | *No* | 144 (29%) | 348 (71%) | 1.00 |  | 205 (54%) | 173 (46%) | 1.00 |  |
|  | *Yes*  *Unknown †* | 13 (45%)  *2* | 16 (55%)  *13* | 1.72 | 0.06 | 15 (65%)  *3* | 8 (35%)  *4* | 1.35 | 0.26 |

* P-value from Wald test accompanying Hazard Ratio in the Cox proportional hazards model

Note: IQR=Inter-quartile range. (a) Univariable model for new *spa*-type acquisition including *S. aureus* CC at recruitment also adjusted for recruitment-negative versus positives. Acquisition and loss models exclude 27 and 19 participants respectively who returned only 1 post-recruitment or post-initial-positive swabs respectively. (b) Total participants analysed fewer than supplementary table 2a as GP record information missing for 23 (4%) and 13 (3%) participants returning ≥2 swabs in analyses of acquisition and loss respectively. **A small number of patients (1-43(1-9%)) for whom particular details of previous healthcare exposure could not be identified from GP records were treated as not having exposure in regression models (in italics in table,** *†***). Inpatient and outpatient exposure included that in the Oxford University Hospitals (OUH) NHS Trust and elsewhere. Time since recent healthcare exposure was truncated at ten years, except for time since more recent GP appointment, which was truncated at five years (approximate 99^th^ percentiles). Where <10% participants had never recorded a healthcare exposure (outpatient, GP or practice nurse appointment, antibiotics; ** in table) median (IQR) time since exposure is provided for those with the exposure, but hazard ratio is estimated considering those without the exposure at the maximum value (ie 5 or 3 years in the past), For all other healthcare exposures (inpatient, surgery, district nurse appointment) univariable models for time since last exposure also adjusted for whether or not the participant had ever had the exposure. One hundred and sixty nine patients had one or more long term illnesses that have been associated with either *S.aureus* carriage or community acquired *S. aureus* infection: (n=number of patients). type 1 diabetes (5), type 2 diabetes (25), asthma/COPD on inhaled steroids (36), history of cancer (24), history of dermatitis or psoriasis (43), most recent BMI >=30 (102), history of drug misuse (4), dialysis (1), cirrhosis (1).** The effect of one of these long-term illnesses was very similar to the effect of the larger group with any long-term illness shown above.

**Supplementary Table 6: Univariable impact of participant risk factors on whether a *S. aureus spa*-type is carried long-term versus intermittently, or whether carriage is never observed versus intermittent**

**(a) Participant characteristics and behaviour at recruitment**

|  | *(Effect in logistic regression model)* | *S. aureus* long-term same *spa*-type carrier (N=137) n (row %) or median (IQR) | Intermittent/ other carrier (N=198) n (row %) or median (IQR) | Never observed as carrier (N=90) n (row %) or median (IQR) | Long-term same *spa*-type versus intermittent univariable Risk Ratio | Long-term versus intermittent *P* value | Never observed versus intermittent univariable Risk Ratio | Never observed versus intermittent *P* value |
| --- | --- | --- | --- | --- | --- | --- | --- | --- |
| *S. aureus* characteristics | | | | | |  |  |  |
| Recruitment- | *Yes* | 2 (1%) | 59 (39%) | 90 (60%) | N/A |  | N/A |  |
| negative | *No* | 135 (49%) | 139 (51%) | 0 (0%) | N/A |  | N/A |  |
| Modal *spa*-CC present | CC30 *(spa-CC021)* | 44 (40%) | 65 (60%) | 0 (0%) | 1.00 | 0.06 | N/A |  |
|  | CC15 *(spa-CC084)* | 14 (29%) | 35 (71%) | 0 (0%) | 0.59 |  |  |  |
|  | *spa-CC002* | 9 (33%) | 18 (67%) | 0 (0%) | 0.74 |  |  |  |
|  | CC22 *(spa-CC005)* | 16 (73%) | 6 (27%) | 0 (0%) | 3.94 |  |  |  |
|  | *spa-CC127* | 6 (33%) | 12 (67%) | 0 (0%) | 0.74 |  |  |  |
|  | *spa-CC160* | 5 (45%) | 6 (55%) | 0 (0%) | 1.23 |  |  |  |
|  | CC8 *(spa-CC024)* | 3 (27%) | 8 (73%) | 0 (0%) | 0.55 |  |  |  |
|  | *Other CC* | 40 (45%) | 48 (55%) | 0 (0%) | 1.23 |  |  |  |
| Participant characteristics | | | | | |  |  |  |
| Age | *Per 10 years* | 61 (47;68) | 50 (25;63) | 59 (40;65) | 1.28 | <0.001 | 1.15 | 0.03 |
| Sex | *Female* | 58 (26%) | 112 (50%) | 53 (24%) | 1.00 |  | 1.00 |  |
|  | *Male* | 79 (39%) | 86 (43%) | 37 (18%) | 1.77 | 0.01 | 0.91 | 0.71 |
| Student | *No* | 127 (35%) | 156 (44%) | 75 (21%) | 1.00 |  | 1.00 |  |
|  | *Yes* | 10 (15%) | 42 (63%) | 15 (22%) | 0.29 | 0.001 | 0.74 | 0.37 |
| Ethnic background | *White British/Irish* | 128 (32%) | 186 (46%) | 86 (22%) | 1.00 | 0.50 | 1.00 | 0.74 |
|  | *Other white* | 8 (44%) | 8 (44%) | 2 (11%) | 1.45 |  | 0.54 |  |
|  | *Other ethnicity* | 1 (14%) | 4 (57%) | 2 (28%) | 0.36 |  | 1.08 |  |
| Participant behaviour | | | | | |  |  |  |
| Current employment | *No* | 67 (30%) | 100 (45%) | 55 (25%) | 1.00 |  | 1.00 |  |
|  | *Yes* | 70 (34%) | 98 (48%) | 35 (17%) | 1.07 | 0.77 | 0.64 | 0.10 |
| Current healthcare | *No* | 113 (35%) | 147 (45%) | 66 (20%) | 1.00 |  | 1.00 |  |
| related employment | *Yes* | 24 (24%) | 51 (52%) | 24 (24%) | 0.61 | 0.08 | 1.05 | 0.87 |
| Number of other household members | *Lives alone* | 25 (44%) | 24 (42%) | 8 (14%) | 1.00 | 0.002 | 1.00 | 0.003 |
|  | *1 household member* | 71 (36%) | 73 (37%) | 55 (28%) | 0.93 |  | 2.26 |  |
|  | *2/3 household members* | 37 (27%) | 79 (58%) | 21 (15%) | 0.45 |  | 0.80 |  |
|  | *4 or more* | 4 (13%) | 20 (67%) | 6 (20%) | 0.19 |  | 0.90 |  |
|  | *Shared accommodation* | 0 (0%) | 2 (100%) | 0 (0%) | N/A |  | N/A |  |
| Number of other household members with healthcare contact | *Zero* | 113 (32%) | 164 (47%) | 73 (21%) | 1.00 | 0.47 | 1.00 | 0.41 |
|  | *One* | 23 (33%) | 29 (42%) | 17 (25%) | 1.15 |  | 1.32 |  |
|  | *Two or more* | 1 (17%) | 5 (83%) | 0 (0%) | 0.29 |  | N/A |  |
| Current participation | *No* | 93 (35%) | 115 (43%) | 60 (22%) | 1.00 |  | 1.00 |  |
| in contact sport | *Yes* | 44 (28%) | 83 (53%) | 30 (19%) | 0.66 | 0.07 | 0.69 | 0.17 |
| Looks after anyone | *No* | 109 (30%) | 170 (37%) | 79 (22%) | 1.00 |  | 1.00 |  |
| with a disability/old age | *Yes* | 28 (42%) | 28 (42%) | 11 (16%) | 1.56 | 0.13 | 0.85 | 0.66 |

**(b) Participant previous healthcare exposure and co-morbidities at recruitment**

|  | *(Effect in multinomial logistic regression model)* | *S. aureus* long-term same *spa*-type carrier (N=136) n (%) or median (IQR) | Intermittent/other carrier (N=192) n (%) or median (IQR) | Never observed as carrier (N=88) n (%) or median (IQR) | Long-term same *spa*-type versus intermittent univariable Risk Ratio | Long-term versus intermittent *P* value | Never observed versus intermittent univariable Risk Ratio | Never observed versus intermittent *P* value |
| --- | --- | --- | --- | --- | --- | --- | --- | --- |
| Previous healthcare exposure | | | | | |  |  |  |
| Ever been an inpatient | *No* | 21 (27%) | 40 (51%) | 17 (22%) | 1.00 |  | 1.00 |  |
|  | *Yes* | 115 (34%) | 152 (45%) | 71 (21%) | 1.44 | 0.22 | 1.10 | 0.77 |
| Days since last in patient episode | *Per year* | 919 (310;2549) | 1105 (394;4266) | 1139 (356;4394) | 0.95 | 0.15 | 1.01 | 0.88 |
| Ever been an outpatient | *No* | 9 (50%) | 7 (39%) | 2 (11%) | 1.00 |  | 1.00 |  |
|  | *Yes* | 127 (32%) | 185 (46%) | 86 (22%) | 1.87 | 0.22 | 0.61 | 0.55 |
| Days since last outpatient appointment * | *Per year* | 289 (83;1087) | 324 (105;909) | 241 (48;1123) | 1.03 | 0.44 | 0.99 | 0.88 |
| Ever had a GP | *No* | 1 (33%) | 2 (67%) | 0 (0%) | 1.00 |  | 1.00 |  |
| appointment | *Yes* | 135 (33%) | 190 (46%) | 88 (21%) | 1.42 | 0.78 | N/A |  |
| Days since last GP appointment * | *Per year* | 55 (22;169) | 67 (28;161) | 85 (23;212) | 1.08 | 0.54 | 0.94 | 0.72 |
| Ever had a practice nurse | *No* | 0 (0%) | 6 (3%) | 0 (0%) | N/A |  | N/A |  |
| appointment | *Yes*  *Unknown†* | 136 (100%)  *0* | 185 (96%)  *1* | 88 (100%)  *0* | N/A |  | N/A |  |
| Days since last practice nurse appointment * | *Per year* | 95 (24;297) | 110 (36;370) | 76 (24;280) | 0.85 | 0.09 | 0.88 | 0.22 |
| Ever had a district nurse | *No* | 117 (33%) | 156 (44%) | 78 (22%) | 1.00 |  | 1.00 |  |
| appointment | *Yes*  *Unknown †* | 19 (29%)  *2* | 36 (55%)  *3* | 10 (15%)  *0* | 0.70 | 0.26 | 0.56 | 0.12 |
| Days since last district nurse appointment | *Per year* | 2335 (651;5548) | 2466 (973;6704) | 1747 (436;5033) | 1.00 | 0.97 | 0.94 | 0.49 |
| Co-morbidities | | | | | |  |  |  |
| Has a long-term illness | *No* | 46 (25%) | 93 (50%) | 48 (26%) | 1.00 |  | 1.00 |  |
|  | *Yes* | 90 (39%) | 99 (43%) | 40 (17%) | 1.84 | 0.009 | 0.78 | 0.34 |
| Ever had chemotherapy | *Yes* | 134 (33%) | 186 (46%) | 83 (21%) | 1.00 |  | 1.00 |  |
|  | *Yes*  *Unknown†* | 2 (15%)  *0* | 6 (46%)  *1* | 5 (38%)  *0* | 0.46 | 0.35 | 1.87 | 0.31 |
| Ever had renal dialysis | *No* | 136 (33%) | 190 (46%) | 88 (21%) | N/A |  | N/A |  |
|  | *Yes* | 0 (0%) | 2 (100%) | 0 (0%) | N/A |  | N/A |  |
| Ever had surgery | *No* | 31 (32%) | 48 (49%) | 19 (19%) | 1.00 |  | 1.00 |  |
|  | *Yes*  *Unknown †* | 105 (33%)  *0* | 144 (45%)  *1* | 69 (22%)  *0* | 1.13 | 0.64 | 1.21 | 0.54 |
| Days since last surgery | *Per year* | 3276 (754;6484) | 2442 (907;5559) | 2206 (485;5668) | 1.03 | 0.45 | 0.97 | 0.44 |
| Ever prescribed oral | *No* | 114 (32%) | 166 (47%) | 74 (21%) | 1.00 |  | 1.00 |  |
| steroids | *Yes*  *Unknown†* | 22 (35%)  *2* | 26 (42%)  *2* | 14 (23%)  *4* | 1.23 | 0.51 | 1.21 | 0.60 |
| Treatment for skin | *No* | 116 (32%) | 170 (47%) | 77 (21%) | 1.00 |  | 1.00 |  |
| conditions in last 30 days | *Yes*  *Unknown* | 20 (38%)  *3* | 22 (42%)  *0* | 11 (21%)  *0* | 1.33 | 0.39 | 1.10 | 0.80 |
|  | *Yes*  *Unknown†* | 90 (34%)  *6* | 122 (46%)  *6* | 56 (21%)  *0* | 1.12 | 0.62 | 1.17 | 0.57 |
| Ever had a catheter | *No* | 108 (35%) | 143 (46%) | 58 (19%) | 1.00 |  | 1.00 |  |
|  | *Yes*  *Unknown†* | 28 (26%)  *8* | 49 (46%)  *5* | 30 (28%)  *0* | 0.76 | 0.30 | 1.51 | 0.14 |
| Ever prescribed | *No* | 7 (44%) | 6 (38%) | 3 (19%) | 1.00 |  | 1.00 |  |
| antibiotics | *Yes*  *Unknown†* | 129 (32%)  *2* | 186 (46%)  *3* | 85 (21%)  *2* | 0.59 | 0.36 | 0.91 | 0.90 |
| Days since last antibiotic prescribed * | *Per year* | 722 (216;2053) | 464 (154;1759) | 537 (168;1790) | 1.04 | 0.19 | 1.00 | 0.94 |
| Had MRSA previously | *No* | 134 (33%) | 191 (46%) | 87 (21%) | 1.00 |  | 1.00 |  |
|  | *Yes*  *Unknown†* | 2 (50%)  *2* | 1 (25%)  *4* | 1 (25%)  *4* | 2.85 | 0.39 | 2.20 | 0.58 |
| Had MSSA previously | *No* | 130 (33%) | 179 (46%) | 84 (21%) | 1.00 |  | 1.00 |  |
|  | *Yes*  *Unknown†* | 6 (26%)  *2* | 13 (57%)  *4* | 4 (17%)  *4* | 0.64 | 0.37 | 0.66 | 0.47 |

Note: IQR=Inter-quartile range; OR=Odds ratio; N/A=Not applicable. Total numbers of participants in table 3b smaller than supplementary table 3a as GP record information not collected for 9 participants (2 persistent *spa*-type, 6 intermittent and 2 non-carriers). **Other details as per Supplementary Table 2 (including *).**

**Supplementary Table 7: Comparison of participants negative at recruitment who were, and were not, followed up.**

|  | Negative and followed | Negative and not followed | P |
| --- | --- | --- | --- |
| N | 129 | 552 |  |
| Age | 58 (41;65) | 59 (45;70) | 0.11 |
| Sex (male) | 46 (36%) | 210 (38%) | 0.69 |
| Ethnicity - *White British* | 126 (98%) | 504 (91%) | 0.019 |
| *Other white* | 3 (2%) | 29 (5%) |  |
| *Other ethnicity* | 0 (0%) | 19 (3%) |  |
| Number household members - *Lives alone* | 11 (9%) | 104 (19%) | 0.026 |
| *1 household member* | 76 (59%) | 259 (47%) |  |
| *2 or 3 household members* | 34 (26%) | 142 (26%) |  |
| *4+ household members* | 7 (5%) | 39 (7%) |  |
| *Lives in a shared house* | 1 (1%) | 8 (1%) |  |
| Employed | 66 (51%) | 254 (46%) | 0.33 |
| Contact sport | 38 (29%) | 163 (30%) | 1 |
